# Supplementary material for: Climate change and its impact on wheat distribution in semi-arid ecosystems: A case study from the Sultanate of Oman
Source: PLoS One. 2025 Jun 17;20(6):e0326198. doi: 10.1371/journal.pone.0326198 (PMC12173393; doi:10.1371/journal.pone.0326198)
Supplement: S1 File — S1. Wheat distribution and inventory data from the most recent Agricultural Census published by the Ministry of Agriculture, Fisheries Wealth and Water Resources of Oman (https://www.mafwr.gov.om/). S2. Bioclimatic datasets in raster formats were utilised to model the spatial distribution of wheat in Oman for the period from 1970 to 2020. S3. Bioclimate datasets in raster formats were used to model and predict the future geographical distribution of wheat in Oman for the period 2021–2040. S4. Bioclimate datasets in raster formats were used to model and predict the future geographical distribution of wheat in Oman for the period 2040–2061. (ZIP) [file pone.0326198.s001.zip › SI/S1.pdf]

S1 File. Bioclimatic datasets in raster formats were utilised to model the spatial distribution of wheat in Oman for the period from 1970 to 2020: (a) temperature seasonality (SD ×100), (b) mean temperature for the coldest quarter, (c) precipitation in the wettest month, (d) precipitation seasonality (coefficient of variation) and (e) precipitation in the coldest quarter

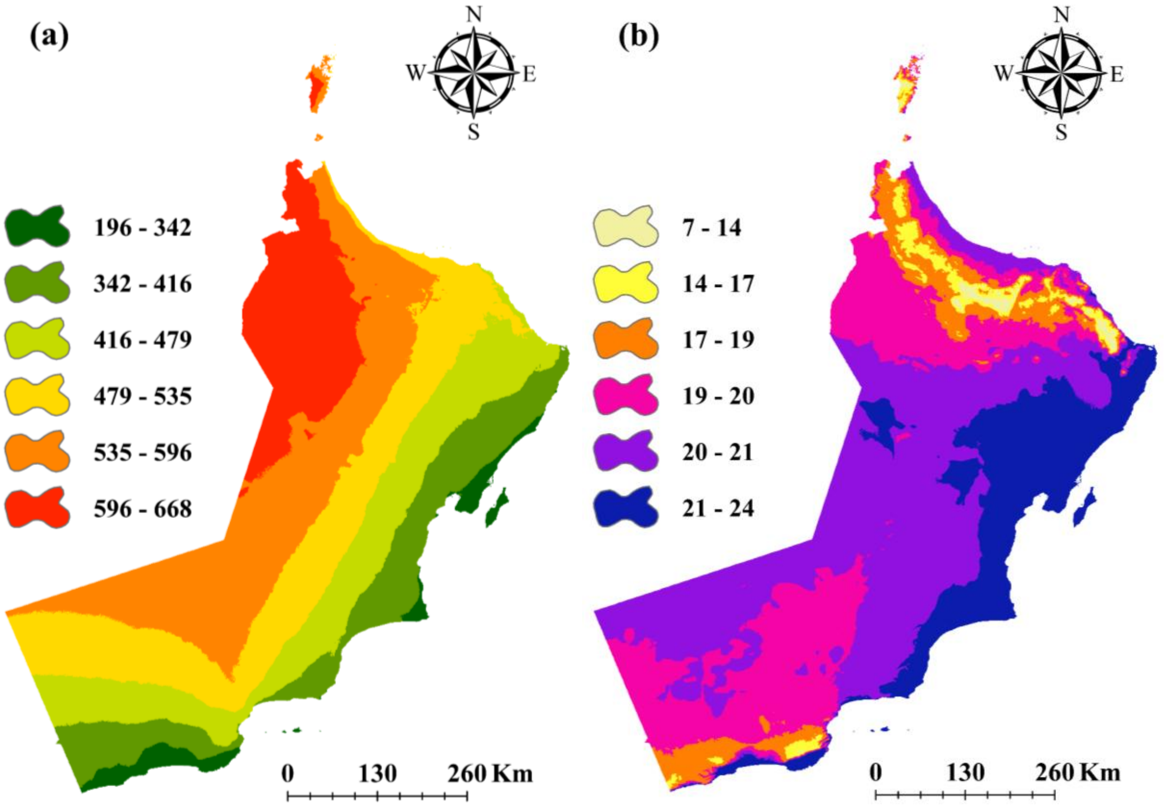

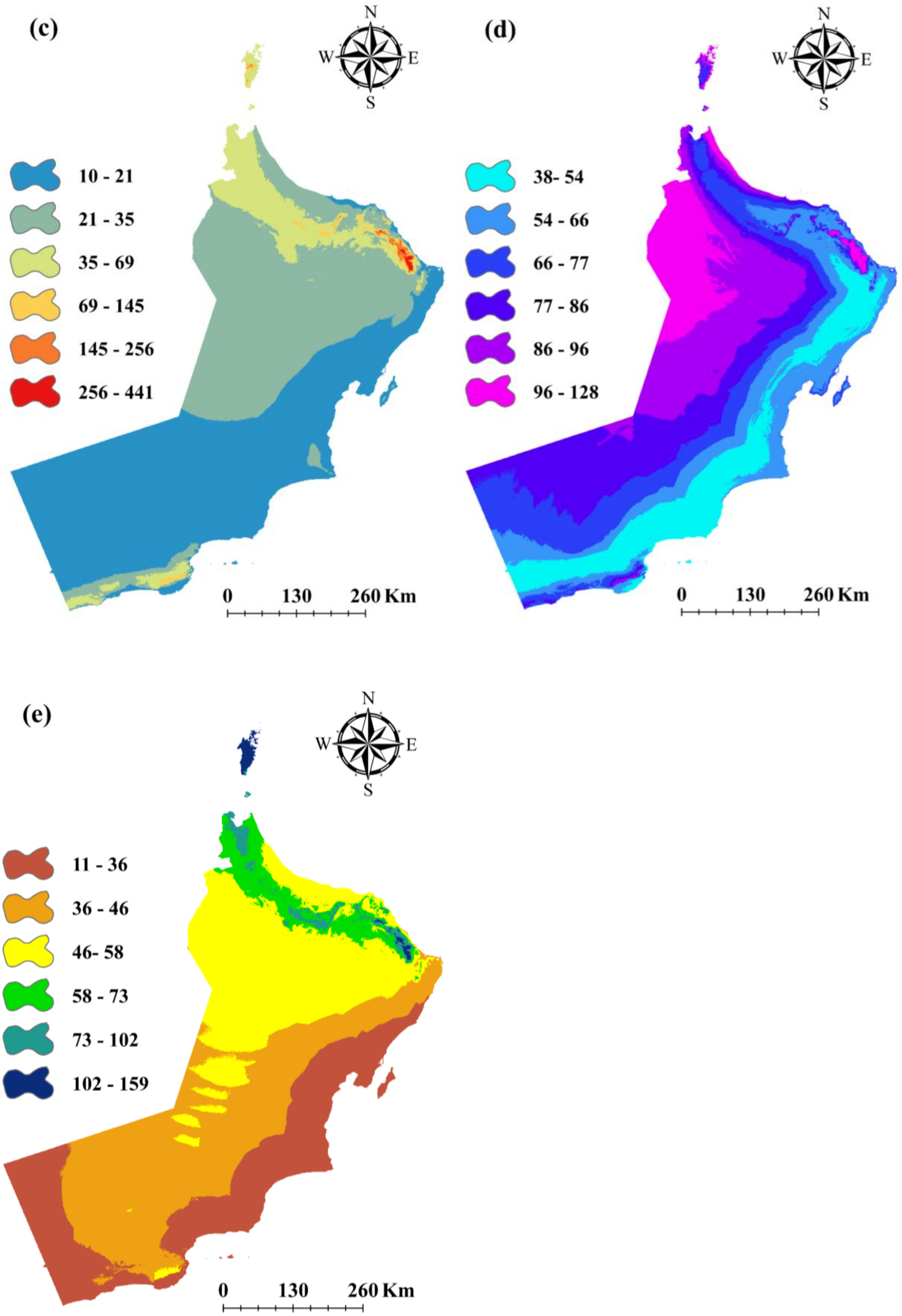
(c)
N
W
E
S
10 - 21
21 - 35
35 - 69
69 - 145
145 - 256
256 - 441
0
130
260 Km
(d)
38- 54
54 - 66
66 - 77
77 - 86
86 - 96
96 - 128
0
130
260 Km
(e)
11 - 36
36 - 46
46- 58
58 - 73
73 - 102
102 - 159
0
130
260 Km
