# Supplementary material for: Climate change and its impact on wheat distribution in semi-arid ecosystems: A case study from the Sultanate of Oman
Source: PLoS One. 2025 Jun 17;20(6):e0326198. doi: 10.1371/journal.pone.0326198 (PMC12173393; doi:10.1371/journal.pone.0326198)
Supplement: S2 File — (PDF) [file pone.0326198.s002.pdf]

ID	Northing	Easting	Elevation	Locations
0	22.83330	57.66670	350	WILAYAT MANAH
1	23.00000	57.30000	500	WILAYAT BAHLA
2	26.17830	56.34190	200	WILAYAT KHASAB
3	26.08330	56.33330	400	WILAYAT KHASAB
4	23.14500	57.67000	1700	WILAYAT NIZWA
5	23.08330	57.13330	500	WILAYAT BAHLA
6	24.06000	56.65000	500	WILAYAT SAHAM
7	23.11330	57.29000	500	WILAYAT AL HAMRA
8	22.16670	59.16670	200	WILAYAT JAALAN BANI BU HASAN
9	23.06610	57.65890	1600	WILAYAT NIZWA
10	23.85000	56.51670	500	WILAYAT SOHAR
11	23.41610	57.13330	700	WILAYAT AR RUSTAQ
12	26.10670	56.18670	1200	WILAYAT KHASAB
13	22.93330	57.53330	400	WILAYAT NIZWA
14	24.28720	56.52530	400	WILAYAT SOHAR
15	24.51810	55.91000	400	WILAYAT MAHADAH
16	25.77000	56.32810	300	WILAYAT DABA
17	23.06530	57.60780	1800	WILAYAT NIZWA
18	23.18080	57.16080	1100	WILAYAT AL HAMRA
19	23.18830	57.05190	700	WILAYAT BAHLA
20	23.13330	57.08330	900	WILAYAT BAHLA
21	23.00000	57.33330	500	WILAYAT BAHLA
22	22.66670	57.33330	400	WILAYAT BAHLA
23	22.83330	58.16670	600	WILAYAT AL MUDAYBI
24	22.63330	58.05000	400	WILAYAT AL MUDAYBI
25	22.83330	57.83330	400	WILAYAT IZKI
26	22.96670	57.53330	400	WILAYAT NIZWA
27	22.90080	57.25360	500	WILAYAT BAHLA
28	23.00000	57.50000	450	WILAYAT NIZWA
29	23.51000	56.50000	400	WILAYAT DANK
30	23.66670	56.50000	500	WILAYAT YANQUL
31	23.50000	56.66670	400	WILAYAT IBRI
32	23.33330	56.66670	300	WILAYAT IBRI
33	22.94330	57.14000	500	WILAYAT BAHLA
34	22.83330	57.33330	400	WILAYAT BAHLA
35	22.95060	57.29220	500	WILAYAT BAHLA
36	23.50000	57.16670	450	WILAYAT AR RUSTAQ
37	24.33330	56.73330	40	WILAYAT SOHAR
38	24.25000	56.50000	142	WILAYAT SOHAR
39	24.40000	56.66670	11	WILAYAT SOHAR
40	24.00000	56.50000	400	WILAYAT SOHAR
41	22.83333	57.50000	400	WILAYAT NIZWA
42	22.16667	59.16667	200	WILAYAT JAALAN BANI BU HASAN
43	24.50000	56.00000	400	WILAYAT MAHADAH
44	25.83333	56.33333	400	WILAYAT DABA
45	23.16667	57.66667	1750	WILAYAT AL AWABI
46	23.16667	57.16667	1100	WILAYAT AL HAMRA
47	23.16667	57.00000	700	WILAYAT BAHLA
48	23.66667	56.50000	300	WILAYAT YANQUL
49	23.50000	56.33333	300	WILAYAT DANK
50	23.00000	57.33333	500	WILAYAT BAHLA
51	22.66667	57.33333	400	WILAYAT BAHLA
52	23.83333	56.33333	500	WILAYAT YANQUL
53	23.33333	57.33333	700	WILAYAT AR RUSTAQ
54	22.81778	58.15583	600	WILAYAT AL MUDAYBI
55	22.50000	58.66667	400	WILAYAT AL QABIL
56	22.50000	58.16667	400	WILAYAT AL MUDAYBI
57	22.93333	57.53333	500	WILAYAT NIZWA
58	22.93333	57.76667	400	WILAYAT IZKI
59	23.05528	57.46833	500	WILAYAT NIZWA
60	23.50000	56.50000	400	WILAYAT DANK

61	23.58556	56.54083	500	WILAYAT YANQUL
62	23.50000	56.66667	400	WILAYAT IBRI
63	23.33333	56.66667	300	WILAYAT IBRI
64	22.83333	57.66667	350	WILAYAT MANAH
65	23.00000	57.16667	500	WILAYAT BAHLA
66	22.83333	57.33333	400	WILAYAT BAHLA
67	23.16667	57.33333	500	WILAYAT AL HAMRA
68	23.50000	57.16667	450	WILAYAT AR RUSTAQ
69	24.16667	56.33333	500	WILAYAT SOHAR
70	24.36667	56.75000	1	WILAYAT SOHAR
71	24.45639	56.65361	16	WILAYAT SOHAR
72	24.41100	56.64200	16	WILAYAT SOHAR
73	23.55190	56.27810	300	WILAYAT DANK
74	23.52780	56.21940	300	WILAYAT DANK
75	22.83333	57.66667	422	WILAYAT MANAH
76	23.85000	56.51667	706	WILAYAT SOHAR
77	22.16667	59.16667	150	WILAYAT JAALAN BANI BU HASAN
78	22.36261	59.17646	0	WILAYAT AL KAMIL WA AL WAFI
79	23.50468	58.35806	0	WILAYAT BAWSHAR
80	23.68643	55.73534	0	WILAYAT AS SUNAYNAH
81	19.27085	57.27318	0	WILAYAT AD DUQM
82	23.60410	58.53015	0	WILAYAT MUTRAH
83	23.36426	58.50618	0	WILAYAT AL AMRAT
84	23.64967	57.86506	0	WILAYAT BARKA
85	18.58420	56.31183	0	WILAYAT AL JAZIR
86	24.71341	56.34693	0	WILAYAT SHINAS
87	21.83446	59.40783	0	WILAYAT JAALAN BANI BU ALI
88	23.22795	58.30347	0	WILAYAT BIDBID
89	22.12659	58.82430	0	WILAYAT BIDIYAH
90	22.77748	58.46798	0	WILAYAT IBRA
91	22.50829	59.42411	0	WILAYAT SUR
92	20.21533	56.23083	0	WILAYAT HAYMA
93	25.28190	56.31878	0	WILAYAT MADHA
94	20.31831	58.70803	0	WILAYAT MASIRAH
95	24.10409	56.23462	0	WILAYAT AL BURAYMI
96	22.63400	59.01730	0	WILAYAT WADI BANI KHALID
97	23.01781	58.66720	0	WILAYAT DAMA WA AT TAIYIN
98	23.46904	58.70043	0	WILAYAT MUSCAT
99	20.45103	57.66203	0	WILAYAT MAHAWT
100	17.22780	54.44360	0	WILAYAT TAQAH
101	17.30678	54.95573	0	WILAYAT SADAH
102	18.39803	53.70502	0	WILAYAT THUMRAYT
103	18.20377	55.27948	0	WILAYAT SHALIM WA JUZOR AL HALLANIYAT
104	17.11871	54.64776	0	WILAYAT MIRBAT
105	16.89084	53.42138	0	WILAYAT RAKHYUT
106	19.31014	54.74251	0	WILAYAT MUQSHIN
107	24.46979	56.40745	0	WILAYAT LIWA
108	18.22422	52.69145	0	WILAYAT AL MAZYUNAH
109	26.10724	56.15478	0	WILAYAT BUKHA
110	16.93345	53.04085	0	WILAYAT DALKUT
111	23.76413	57.26682	0	WILAYAT AS SUWAYQ
112	23.58111	58.18470	0	WILAYAT AS SEEB
113	23.12612	58.90332	0	WILAYAT QURAYYAT
114	17.01670	54.10000	178	WILAYAT SALALAH
115	17.01750	54.08278	14	WILAYAT SALALAH
116	23.50564	57.83177	0	WILAYAT WADI AL MAAWIL
117	23.33606	57.83389	0	WILAYAT NAKHAL
118	17.01667	54.10000	178	WILAYAT SALALAH
119	23.19371	58.03314	0	WILAYAT SAMAIL
120	23.68988	57.57373	0	WILAYAT AL MUSANAAH
121	23.79597	57.00932	0	WILAYAT AL KHABURAH
122	21.70486	57.34226	0	WILAYAT ADAM
