# Supplementary material for: Climate change and its impact on wheat distribution in semi-arid ecosystems: A case study from the Sultanate of Oman
Source: PLoS One. 2025 Jun 17;20(6):e0326198. doi: 10.1371/journal.pone.0326198 (PMC12173393; doi:10.1371/journal.pone.0326198)
Supplement: Attachment — Submitted filename: 568e5e30-a681-4193-82e9-45fb719f2265.pdf [file pone.0326198.s003.pdf]

PLOS ONE

Climate Change and its Impact on Wheat Distribution in Semi-arid Ecosystems

--Manuscript Draft--

**Manuscript Number:**	PONE-D-24-57793
**Article Type:**	Research Article
**Full Title:**	Climate Change and its Impact on Wheat Distribution in Semi-arid Ecosystems
**Short Title:**	Wheat Distribution in Semi-arid Ecosystems
**Corresponding Author:**	Khalifa Alkindi, PhD<br>University of Nizwa<br>Nizwa, OMAN
**Keywords:**	Oman; climate change; wheat; maximum entropy; suitability
**Abstract:**	The Sultanate of Oman, located in the south-eastern Arabian Peninsula, is rich in natural resources, including oil and natural gas, which play a crucial role in its economy. Climate change refers to long-term alterations in weather patterns, including temperature, precipitation and extreme events, typically observed over a 30-year period. The primary aim of this study is to mitigate the impact of climate change on wheat production in Oman by identifying optimal cultivation areas over the next four decades. the maximum entropy (MaxEnt) model was used to predict the suitability of wheat cultivation in Oman across four distinct time periods: 1970–2020, 2021–2040, 2041–2060 and 2061–2080. The MaxEnt model effectively predicted wheat distribution in the study region, with strong outcomes across all time periods. In the reference period (1970–2020), the model achieved an area under curve (AUC) score of 0.82, indicating high precision in identifying regions suitable for wheat cultivation. For 2021–2040, the AUC decreased slightly to 0.81, reflecting potential changes in environmental conditions that could affect wheat distribution patterns. In 2041–2060, the AUC returned to 0.82, maintaining strong predictive accuracy despite projected climate change impacts. After 2061, the AUC increased slightly to 0.83, potentially indicating that wheat distribution patterns become more distinct under projected climate scenarios or that the model's environmental factors gain greater importance as climate change progresses. These results underscore the MaxEnt model's effectiveness in predicting wheat distribution amidst climate change, providing valuable insights into long-term agricultural planning and sustainable practices. Given that various locations in Oman have historically cultivated wheat, identifying the most appropriate regions for future production under climate change is critical. To support strategic decision-making, further studies on this topic are essential.
**Order of Authors:**	Khalifa Alkindi, PhD
	Ali Hussain Al-Lawati
**Additional Information:**	
**Question**	**Response**
**Financial Disclosure**<br><br>Enter a financial disclosure statement that describes the sources of funding for the work included in this submission. Review the submission guidelines for detailed requirements. View published research articles from *PLOS ONE* for specific examples.<br><br>This statement is required for submission and **will appear in the published article** if	The author(s) received no specific funding for this work.

Powered by Editorial Manager® and ProduXion Manager® from Aries Systems Corporation

the submission is accepted. Please make sure it is accurate.<br><br>**Funded studies**<br>Enter a statement with the following details:<br>• Initials of the authors who received each award<br>• Grant numbers awarded to each author<br>• The full name of each funder<br>• URL of each funder website<br>• Did the sponsors or funders play any role in the study design, data collection and analysis, decision to publish, or preparation of the manuscript?<br><br>Did you receive funding for this work?	
**Competing Interests**<br><br>Use the instructions below to enter a competing interest statement for this submission. On behalf of all authors, disclose any competing interests that could be perceived to bias this work—acknowledging all financial support and any other relevant financial or non-financial competing interests.<br><br>This statement is required for submission and **will appear in the published article** if the submission is accepted. Please make sure it is accurate and that any funding sources listed in your Funding Information later in the submission form are also declared in your Financial Disclosure statement.<br><br>View published research articles from *PLOS ONE* for specific examples.	The authors have declared that no competing interests exist.

**NO authors have competing interests**<br><br>Enter: *The authors have declared that no competing interests exist.*<br><br>**Authors with competing interests**<br><br>Enter competing interest details beginning with this statement:<br><br>*I have read the journal's policy and the authors of this manuscript have the following competing interests: [insert competing interests here]*<br><br>* typeset	
**Ethics Statement**<br><br>Enter an ethics statement for this submission. This statement is required if the study involved:<br><br>• Human participants<br>• Human specimens or tissue<br>• Vertebrate animals or cephalopods<br>• Vertebrate embryos or tissues<br>• Field research<br><br>Write "N/A" if the submission does not require an ethics statement.<br><br>General guidance is provided below. Consult the submission guidelines for detailed instructions. **Make sure that all information entered here is included in the Methods section of the manuscript.**	"N/A" if the submission does not require an ethics statement.

Powered by Editorial Manager® and ProduXion Manager® from Aries Systems Corporation

**Format for specific study types**<br><br>**Human Subject Research (involving human participants and/or tissue)**<br>• Give the name of the institutional review board or ethics committee that approved the study<br>• Include the approval number and/or a statement indicating approval of this research<br>• Indicate the form of consent obtained (written/oral) or the reason that consent was not obtained (e.g. the data were analyzed anonymously)<br><br>**Animal Research (involving vertebrate animals, embryos or tissues)**<br>• Provide the name of the Institutional Animal Care and Use Committee (IACUC) or other relevant ethics board that reviewed the study protocol, and indicate whether they approved this research or granted a formal waiver of ethical approval<br>• Include an approval number if one was obtained<br>• If the study involved *non-human primates*, add *additional details* about animal welfare and steps taken to ameliorate suffering<br>• If anesthesia, euthanasia, or any kind of animal sacrifice is part of the study, include briefly which substances and/or methods were applied<br><br>**Field Research**<br><br>Include the following details if this study involves the collection of plant, animal, or other materials from a natural setting:<br>• Field permit number<br>• Name of the institution or relevant body that granted permission	
**Data Availability**<br><br>Authors are required to make all data underlying the findings described fully available, without restriction, and from the time of publication. PLOS allows rare exceptions to address legal and ethical concerns. See the PLOS Data Policy and FAQ for detailed information.	Yes - all data are fully available without restriction

Powered by Editorial Manager® and ProduXion Manager® from Aries Systems Corporation

A Data Availability Statement describing where the data can be found is required at submission. Your answers to this question constitute the Data Availability Statement and **will be published in the article**, if accepted.<br><br>**Important:** Stating 'data available on request from the author' is not sufficient. If your data are only available upon request, select 'No' for the first question and explain your exceptional situation in the text box.<br><br>Do the authors confirm that all data underlying the findings described in their manuscript are fully available without restriction?	
**Describe where the data may be found in full sentences. If you are copying our sample text, replace any instances of XXX with the appropriate details.**<br><br>• If the data are **held or will be held in a public repository**, include URLs, accession numbers or DOIs. If this information will only be available after acceptance, indicate this by ticking the box below. For example: *All XXX files are available from the XXX database (accession number(s) XXX, XXX.).*<br>• If the data are all contained **within the manuscript and/or Supporting Information files**, enter the following: *All relevant data are within the manuscript and its Supporting Information files.*<br>• If neither of these applies but you are able to provide **details of access elsewhere**, with or without limitations, please do so. For example:<br><br>*Data cannot be shared publicly because of [XXX]. Data are available from the XXX Institutional Data Access / Ethics Committee (contact via XXX) for researchers who meet the criteria for access to confidential data.*<br><br>*The data underlying the results presented in the study are available from (include the name of the third party*	Data will be provided as supporting information documents

and contact information or URL).<br>• This text is appropriate if the data are owned by a third party and authors do not have permission to share the data.<br><br>* typeset	
Additional data availability information:	Tick here if the URLs/accession numbers/DOIs will be available only after acceptance of the manuscript for publication so that we can ensure their inclusion before publication.

Click here to access/download;Manuscript;Khalifa and Ali.docx

Climate Change and its Impact on Wheat Distribution in Semi-arid Ecosystems

Khalifa M. Al-Kindi[1]* and Ali Hussain Al-Lawati[2]

[1]UNESCO Chair of Aflaj Studies, Archaeohydrology, University of Nizwa, Nizwa, Sultanate Oman

[2]Natural and Medical Sciences Research Center, University of Nizwa, Nizwa, Sultanate of Oman

Khalifa M. Al-Kindi*

alkindi.k@unizwa.edu.om

Abstract

The Sultanate of Oman, located in the south-eastern Arabian Peninsula, is rich in natural resources, including oil and natural gas, which play a crucial role in its economy. Climate change refers to long-term alterations in weather patterns, including temperature, precipitation and extreme events, typically observed over a 30-year period. The primary aim of this study is to mitigate the impact of climate change on wheat production in Oman by identifying optimal cultivation areas over the next four decades. the maximum entropy (MaxEnt) model was used to predict the suitability of wheat cultivation in Oman across four distinct time periods: 1970–2020, 2021–2040, 2041–2060 and 2061–2080. The MaxEnt model effectively predicted wheat distribution in the study region, with strong outcomes across all time periods. In the reference period (1970–2020), the model achieved an area under curve (AUC) score of 0.82, indicating high precision in identifying regions suitable for wheat cultivation. For 2021–2040, the AUC decreased slightly to 0.81, reflecting potential changes in environmental conditions that could affect wheat distribution patterns. In 2041–2060, the AUC returned to 0.82, maintaining strong predictive accuracy despite projected climate change impacts. After 2061, the AUC increased slightly to 0.83, potentially indicating that wheat distribution patterns become more distinct under projected climate scenarios or that the model's environmental factors gain greater importance as climate change progresses. These results underscore the MaxEnt model's effectiveness in predicting wheat distribution amidst climate change, providing valuable insights into long-term agricultural planning and sustainable practices. Given that various locations in Oman have historically cultivated wheat, identifying the most appropriate regions for future production under climate change is critical. To support strategic decision-making, further studies on this topic are essential.

Key words: Oman, climate change, wheat, maximum entropy, suitability

1. Introduction

The Sultanate of Oman, located in the south-eastern Arabian Peninsula, is rich in natural resources, including oil and natural gas, which play a crucial role in its economy [1]. Its diverse geography supports significant biodiversity, with landscapes ranging from mountains and deserts to a long coastline [2]. Understanding Oman's geography and climate is vital for agricultural studies and assessing the impacts of climate change [3,4].

Climate change refers to long-term alterations in weather patterns, including temperature, precipitation and extreme events, typically observed over a 30-year period. It is primarily driven by greenhouse gas (GHG) emissions such as carbon dioxide, methane, nitrous oxides and chlorofluorocarbons [5]. While natural systems contribute to climate change, human activities are the dominant cause [6]. Its impacts span environmental and economic domains, significantly affecting food production, especially in developing countries [7].

Studies reveal that climate change threatens food security by affecting agricultural systems. It reduces livestock productivity, increases mortality and degrades soil health through erosion, nutrient runoff and loss of soil quality. For plants, it alters phenological development, productivity and biomass and reduces chilling hours for perennials and growing degree days Hatfield et al. (2020). Bajwa et al. (2020) have highlighted the detrimental impact of pest infestations on wheat production, underscoring the compounded challenges facing agriculture.

Despite Oman's dependence on agriculture and its vulnerability to climate change, limited research explores the spatial distribution of wheat and its interaction with bioclimatic factors. Previous studies overlook Oman’s unique climatic zones and the specific challenges faced by its agricultural sector [10,11]. Generalisations from global studies on wheat modelling fail to capture the nuanced bioclimatic conditions in Oman [12].

The absence of research specifically tailored to Oman’s diverse climatic zones—coastal, mountainous and desert—presents a clear gap in understanding the impacts of these varied climates on wheat cultivation [13]. Additionally, limited investigations linking bioclimatic variables to wheat distribution have hindered the development of localised insights crucial for

effective agricultural planning [14]. Factors such as temperature and precipitation, which significantly influence the spatial distribution of wheat, are especially important in regions such as Oman, where water scarcity and climate variability are pressing challenges [15].

The lack of predictive modelling for future wheat distribution in Oman represents a substantial research gap [16]. Forecasting how wheat distribution patterns will evolve under climate change is critical for adaptive agricultural strategies. Without such studies, policymakers and stakeholders lack the essential information required for resilient and sustainable agricultural practices.

To address these gaps, targeted research is needed that integrates tools such as geographical information systems, remote sensing and predictive modelling techniques like maximum entropy (MaxEnt) to analyse wheat distribution in Oman [17]. The MaxEnt model is particularly effective for understanding how climate change influences wheat cultivation potential [18–20]. For instance, Zhao et al. (2024) applied MaxEnt to evaluate potential wheat distribution under 1.5°C and 2°C global warming scenarios, revealing that climate change would significantly affect planting suitability, with over half of the areas experiencing shifts due to rising temperatures. Similar studies in Oman could address current knowledge deficits, offering valuable insights into sustainable agriculture and climate adaptation [22,23].

The primary aim of this study is to mitigate the impact of climate change on wheat production in Oman by identifying optimal cultivation areas over the next four decades. This information will aid the government and investors in making informed decisions regarding suitable locations for wheat cultivation. While challenges in wheat farming in Oman have been acknowledged (Farooq et al., 2022), there is no comprehensive data on areas most suitable for wheat production under changing climatic conditions. Given that various locations in Oman have historically cultivated wheat, identifying the most appropriate regions for future production under climate change is critical. To support strategic decision-making, further studies on this topic are essential.

2. Materials and Methods

2.1. Study Area

Oman is located on the south-eastern edge of the Arabian Peninsula, bordered by the Arabian Sea to the southeast, the Sea of Oman to the northeast and the United Arab Emirates and Saudi Arabia to the west. Additionally, Oman shares a maritime boundary with Iran and is strategically

positioned near the Strait of Hormuz to the north. The country's geography is marked by significant diversity, encompassing vast mountain ranges, expansive deserts and a lengthy coastline. Notably, the Al Hajar mountain range runs through the northern part of the country (Figure 1), while the Rub' al Khali, or Empty Quarter, one of the world's largest uninterrupted sand deserts, stretches across Oman's southwest.

Oman's climate varies considerably, from arid desert conditions in the lowlands to more temperate climates in the higher elevations of its mountains. Coastal regions, including the capital city, Muscat, typically experience a hot desert climate with elevated temperatures year-round. In contrast, the mountainous areas benefit from a milder and cooler climate, offering a more temperate environment. Spanning an area of 309,500 km^2, Oman's agricultural sector covers approximately 14,662 km^2, which constitutes about 4.7% of the total land area. The majority of the agricultural land consists of permanent meadows and pastures (92.1%), followed by arable land (5.6%) and permanent crops (2.3%) (https://wsp.mafwr.gov.om/).

In the study area focused on wheat cultivation, a random point extension was used in ArcGIS Pro 3.3 to model the bioclimate factors. A total of 110 points were generated, with the dataset randomly divided into two portions: 70% for training the model and 30% for validation, as illustrated in Figure 1.

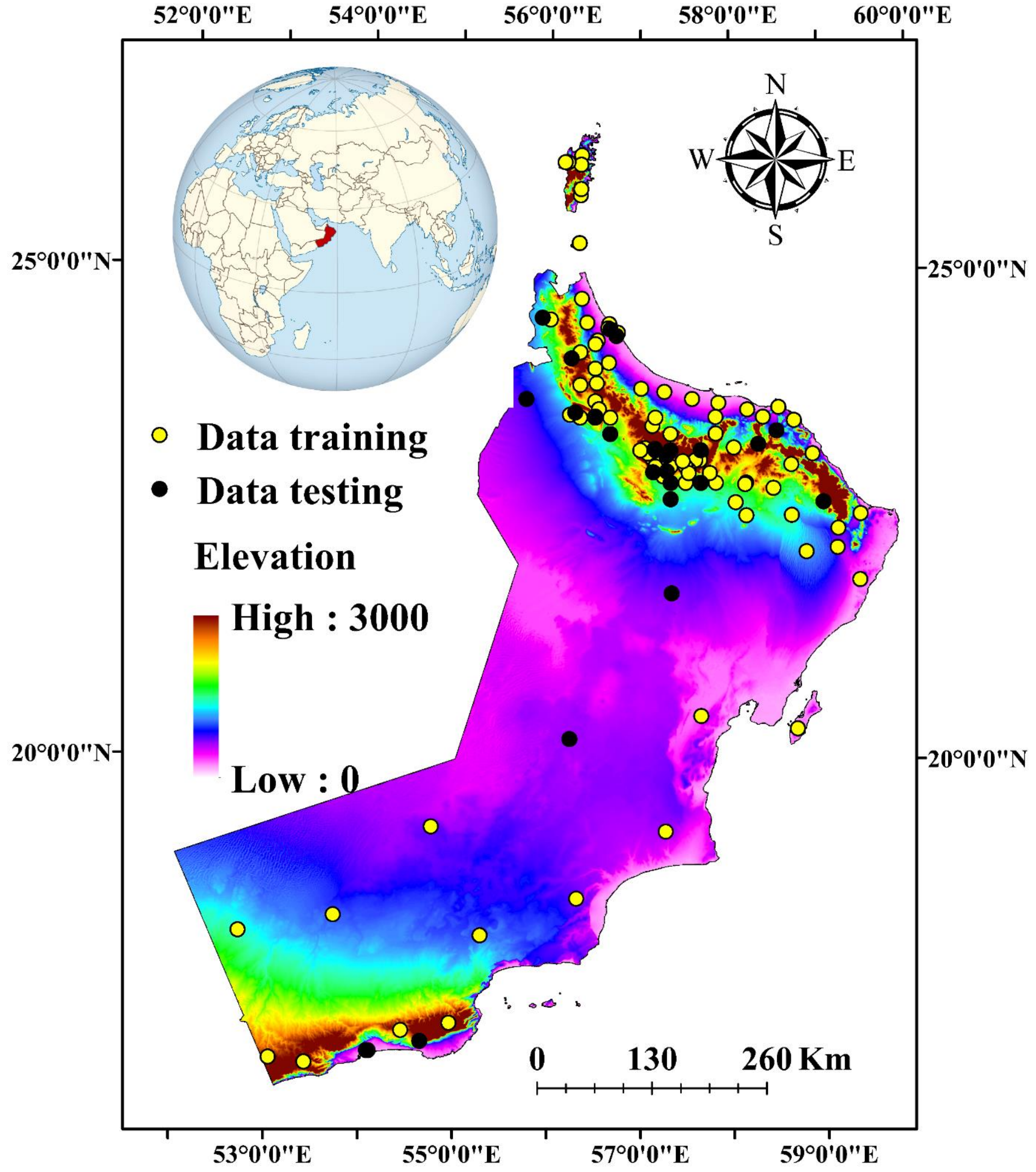

Figure 1. Locations within the study area, featuring the elevation map and wheat data used for training and testing the model.

2.2. Data Collection and Spatial Processing

Wheat distribution and inventory data were sourced from the latest Agricultural Census, as published by the Ministry of Agriculture, Fisheries Wealth & Water Resources of Oman (accessed

on 22 March, 2013). In Oman, the wheat-growing season spans November to April. Key factors influencing wheat production include temperature, water availability (especially soil moisture), humidity and wind conditions and solar radiation. Due to Oman's predominantly arid climate, irrigation systems, rather than natural precipitation, largely fulfil the water requirements for wheat cultivation.

Herein, a range of bioclimatic factors were assessed, including annual mean temperature, temperature seasonality, minimum temperature of the coldest month, mean temperature of the coldest quarter, annual precipitation, precipitation during the wettest month and precipitation seasonality. These bioclimatic variables were obtained from the WorldClim database (https://www.worldclim.org/), a global repository of high-resolution climate and meteorological data. The WorldClim dataset is well-suited for spatial modelling and cartographic applications due to its high spatial resolution. Table 1 provides an overview of the critical bioclimatic variables deemed essential for predicting the suitability of areas for wheat production.

Table 1. Bioclimatic factors identified for wheat production in Oman.

Factor	Definition
**BIO1**	Annual Mean Temperature
**BIO4**	Temperature Seasonality (standard deviation ×100)
**BIO6**	Min Temperature of Coldest Month
**BIO11**	Mean Temperature of Coldest Quarter
**BIO12**	Annual Precipitation
**BIO13**	Precipitation of Wettest Month
**BIO15**	Precipitation Seasonality (Coefficient of Variation)
**BIO16**	Precipitation of Wettest Quarter
**BIO19**	Precipitation of Coldest Quarter

2.3. Multicollinearity Analysis

To evaluate the influence of specific variables on the accuracy of the final wheat distribution maps, a multicollinearity analysis was conducted using variance inflation factors (VIFs). This technique helps identify instances where multiple variables provide redundant information. Any variable

with a VIF exceeding the threshold of 7.5 was excluded from further analysis to avoid multicollinearity [25–27]. The machine learning process began with the application of the VIF analysis to assess the inter-correlation among the nine bioclimatic variables selected based on their relevance to the theoretical framework (Table 1).

2.4. Maximum Entropy Method

In this study, the MaxEnt model was used to predict the suitability of wheat cultivation in Oman across four distinct time periods: 1970–2020, 2021–2040, 2041–2060 and 2061–2080. This approach incorporated both historical and projected bioclimatic data to evaluate how environmental changes could influence wheat distribution. The MaxEnt method is a widely used machine-learning technique for species distribution modelling that also applies to predicting wheat suitability under various environmental scenarios [28–30]. The underlying principle of MaxEnt is based on maximum entropy theory, which posits that when estimating a probability distribution, the distribution should be chosen to maximise entropy, subject to the given constraints [31–34]. This approach ensures that the model's conclusions are based solely on the data without introducing any unwarranted assumptions or biases.

2.5 Evaluating the MaxEnt Model

To evaluate the MaxEnt model's effectiveness, the area under the curve (AUC) was used to measure its ability to distinguish between species presence locations and background points based on environmental suitability [35,36]. The assessment involves generating a receiver operating characteristic curve, which illustrates the relationship between the true and false positive rates across various probability thresholds. The AUC provides a single value summarising the model's performance, ranging from 0.5 (random prediction) to 1 (perfect prediction) [37,38]. Models with an AUC above 0.7 are considered effective. For instance, an AUC of 0.85 indicates an 85% likelihood of correctly ranking presence points above background points. This metric offers a reliable numerical assessment of the MaxEnt model's accuracy in predicting species distribution using environmental data [39]. Figure 2 outlines the methodology used to evaluate wheat cultivation suitability in Oman, incorporating both historical and projected bioclimatic factors in a step-by-step analysis.

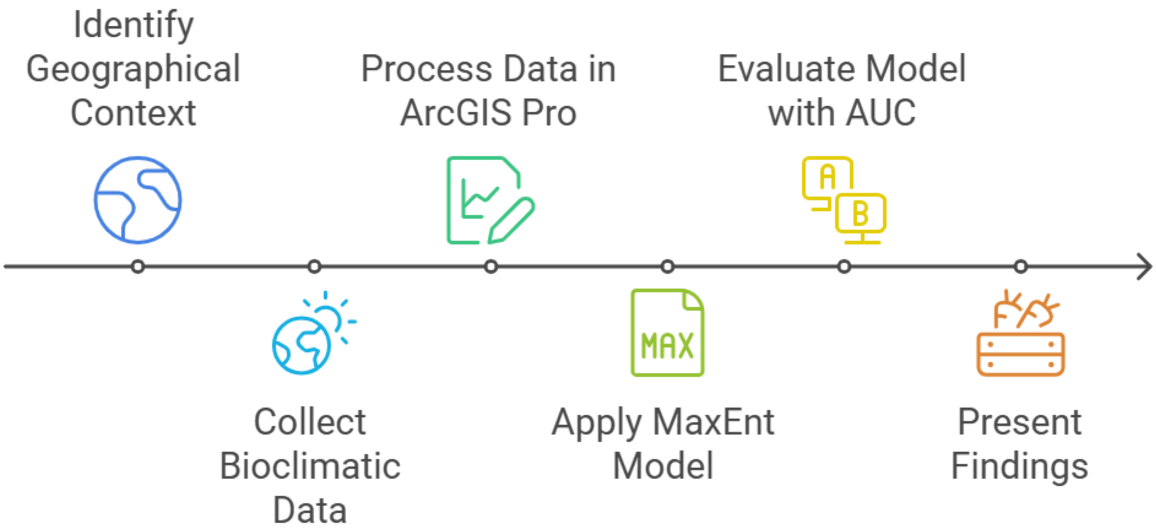

Figure 2. Schematic representation of the sequential steps in the analytical process.

3. Results and Discussion

3.1. Multicollinearity Analysis

To identify variables providing redundant information that could affect the accuracy of the final wheat distribution maps, a multicollinearity analysis was conducted using VIFs [40]. Variables exceeding the 7.5 threshold were excluded from further analysis [41]. Following this assessment, five of the nine variables were deemed suitable for inclusion in the MaxEnt model and successfully passed the VIF test. The selected variables were temperature seasonality (SD ×100), mean temperature of the coldest quarter, precipitation of the wettest month, precipitation seasonality and precipitation of the coldest quarter. The inclusion of these variables enabled the model to accurately predict the geographical distribution of wheat (Table 2).

Table 2. Selected variables used in the model to predict the geographical distribution of wheat.

Factors	VIF
**Annual Mean Temperature**	8.7
**Temperature Seasonality (SD ×100)**	2.61
**Min Temperature of Coldest Month**	9.1
**Mean Temperature of Coldest Quarter**	4.10
**Annual Precipitation**	10.2

**Precipitation of Wettest Month**	5.05
**Precipitation Seasonality**	1.55
**Precipitation of Wettest Quarter**	12.01
**Precipitation of Coldest Quarter**	3.06

3.2. Maximum Entropy Method

3.2.1. Model's Evaluation

The MaxEnt model effectively predicted wheat distribution in the study region, with strong outcomes across all time periods. In the reference period (1970–2020), the model achieved an AUC score of 0.82, indicating high precision in identifying regions suitable for wheat cultivation (Figure 3). This demonstrates the model's success in capturing wheat distribution patterns during this period. Guo et al. (2024) also recognised the MaxEnt model as effective for forecasting wheat cultivation trends, especially regarding climate change adaptation and agricultural strategy development.

For 2021–2040, the AUC decreased slightly to 0.81 (Figure 3), reflecting potential changes in environmental conditions that could affect wheat distribution patterns. Despite this, the model remained robust in its predictive ability. In 2041–2060, the AUC returned to 0.82, maintaining strong predictive accuracy despite projected climate change impacts [43].

The AUCs remained consistent throughout these periods, suggesting that the model's bioclimatic variables are effective in predicting wheat-growing potential. After 2061, the AUC increased slightly to 0.83, potentially indicating that wheat distribution patterns become more distinct under projected climate scenarios or that the model's environmental factors gain greater importance as climate change progresses. These results underscore the MaxEnt model's effectiveness in predicting wheat distribution amidst climate change, providing valuable insights into long-term agricultural planning and sustainable practices [18,19].

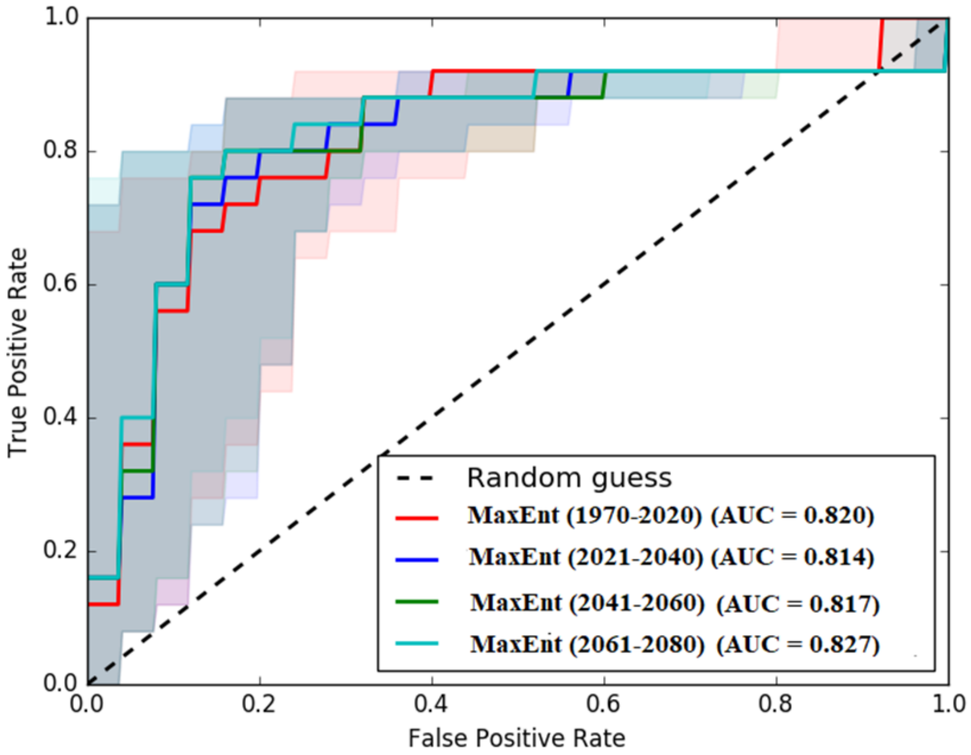

Figure 3. Model assessment showing AUC outcomes based on historical and projected bioclimatic variables.

3.2.2. Temporal analysis of wheat cultivation suitability areas

The temporal assessment of suitability zones by the MaxEnt model (1970–2080) reveals distinct patterns in both percentage and spatial extent (km^2) across five suitability categories: extremely high, high, medium, low and extremely low. Figure 4 presents wheat-growing suitability maps generated using the MaxEnt approach. These changes offer valuable insights into the evolution of agricultural potential, with significant implications for land-use planning and agricultural policy development [44].

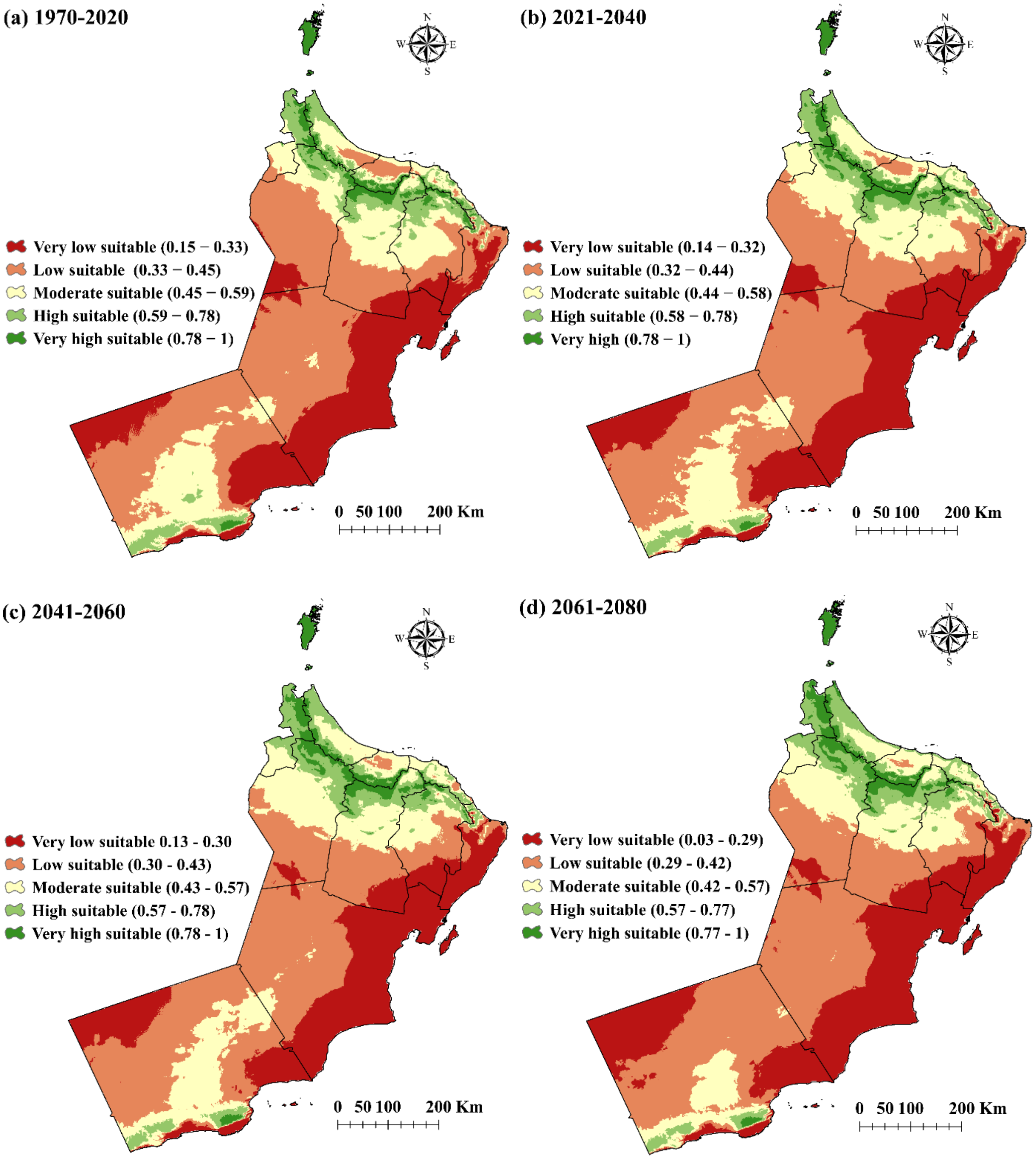

Figure 4. MaxEnt-generated wheat suitability maps.

Regions with optimal environmental conditions for wheat growth are classified as areas with very high suitability. Between 1970 and 2020, only 3% of the total area, approximately 9,285 km^2, was deemed highly suitable for wheat farming. However, by 2041–2060, this classification saw a slight increase to 4%, expanding to 12,380 km^2. This level of suitability remained stable through 2061–2080 (Figures 5a and 5b). This modest increase suggests that certain environmental factors, such

as rainfall patterns, soil quality, or temperature variations, may have improved, enhancing land suitability for wheat cultivation.

Despite this expansion, the total area of very high suitability remains small compared to other suitability categories, indicating that only a limited fraction of land is highly suitable for wheat farming. These optimal zones should be prioritised for the implementation of innovative farming techniques, including precision agriculture, to maximise yields and ensure sustainable wheat production [45,46]. The areas classified as highly suitable for wheat cultivation remained constant across all time periods, covering 8% of the total land area, approximately 24,760 km^2. This stability suggests that the environmental conditions necessary for moderately optimal wheat growth are resilient to climate or ecological changes (Figures 4a and 4b). These regions, which maintain their extent, consistently qualify as highly suitable, although they may yield less than the very highly suitable areas. Ensuring long-term wheat farming productivity in these areas requires prudent management of water and soil resources.

The intermediate suitability zones, which accounted for 20% of the total area (about 61,900 km^2) from 1970 to 2020, displayed greater variability. This proportion remained stable until 2040 but increased slightly to 22% (about 68,090 km^2) during 2041–2060 (Figures 4a and 4b). Projections indicate a decline to 16% (approximately 49,520 km^2) by 2061–2080. This fluctuation highlights the vulnerability of these regions to changes in climate and environmental conditions. The temporary increase in moderately suitable areas during 2041–2060 may be due to favourable weather patterns or improved land management practices. However, the subsequent decline suggests growing challenges, possibly arising from rising temperatures, soil degradation or decreasing water availability. The reduction in moderately suitable land underscores the increasing difficulty of wheat production in these areas without significant interventions, such as the use of drought-resistant wheat cultivars or advanced irrigation systems [47].

Areas with low suitability consistently dominate the landscape, accounting for 40%–43% of the total area throughout the study period. In concrete terms, this corresponds to approximately 133,085 km^2 during 1970–2020 and 2021–2040. During 2041–2060, this area slightly decreases to 123,800 km^2, before returning to 133,085 km^2 during 2061–2080 (Figures 4a and 4b). The persistence of this classification indicates that a significant portion of the land is only marginally suitable for wheat production, likely due to factors such as poor soil conditions, limited water

resources or an unsuitable climate. The small decrease in low-suitability areas during the mid-century period may reflect temporary improvements in certain environmental conditions. However, these enhancements are insufficient to elevate large areas to higher suitability classifications. To make wheat cultivation feasible in these low-suitability areas in the future, substantial investments in technologies such as advanced irrigation systems or soil improvement methods will be necessary [48].

A comparison of percentage-and area-based statistics reveals the extent of changes in land suitability and their geographical implications for wheat production. Although the proportion of areas classified as very high and high suitability remained relatively constant or saw slight increases, their actual land coverage remained limited, indicating that only a small fraction of the total area is highly productive (Figures 4a and 4b). To optimise yields and ensure food security, these regions should be prioritised for wheat cultivation. The fluctuations in moderately suitable areas, coupled with the continued dominance of low and very low suitability zones, highlight the challenges that climate change and environmental degradation pose to agricultural practices [49]. The contraction of moderately suitable regions and the expansion of areas with very low suitability emphasise the need for adaptable management strategies. These strategies should include farming methods resilient to climate change, techniques for improving soil quality and the development of efficient water management infrastructure to sustain agricultural output amidst evolving climatic conditions [50].

Furthermore, the slight increase in very low suitability areas towards the end of the century suggests the necessity for long-term planning and policies that address the broader impacts of climate change. This could involve diversifying crops, adjusting land-use patterns and enhancing land management practices to build resilience in low-suitability regions [44,51].

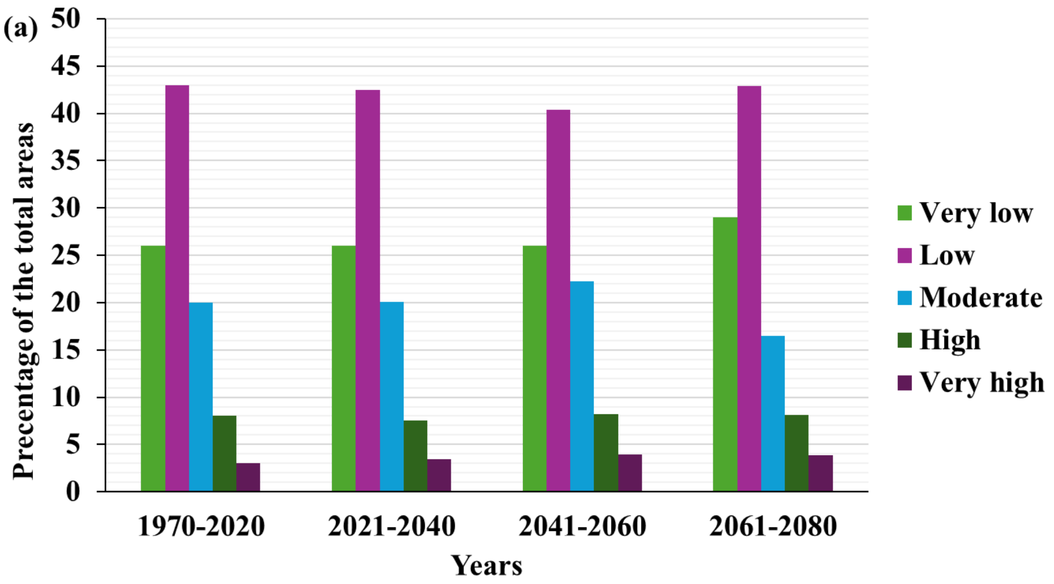

Figure 5a. Temporal wheat cultivation suitability as a percentage, showing the land distribution across five categories—very high, high, moderate, low and very low—from 1970 to 2080 based on MaxEnt model projections.

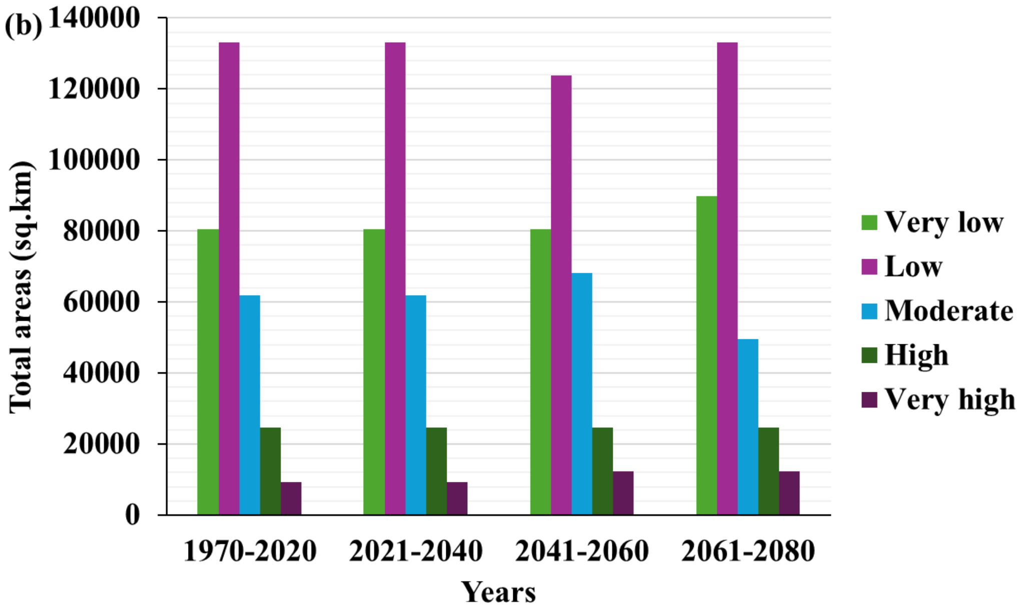

Figure 5b. Temporal suitability of wheat cultivation by area (km^2). This chart illustrates the area of land (in km^2) classified into five suitability categories—very high, high, moderate, low and very low—for wheat cultivation from 1970 to 2080 based on MaxEnt model projections.

3.5. Wheat versus Bioclimate Factors (1970–2020)

3.5.1. Wheat probability versus temperature seasonality

The inverse correlation between wheat distribution and temperature seasonality (SD ×100) is illustrated in Figure 5a using the AUC. Regions with consistent temperatures are more conducive to wheat growth, as demonstrated by the high occurrence probability (exceeding 0.99%) in areas where temperature seasonality is below 200°C. However, this probability declines as seasonality exceeds 300°C, with the curve plateauing at higher values. The likelihood of the presence of wheat drops sharply above 400°C (SD ×100), indicating that increased temperature fluctuations reduce the suitability for wheat cultivation [52]. This highlights wheat's vulnerability to unstable climatic conditions (Figure 6a).

3.5.2. Wheat probability versus the mean temperature of the coldest quarter

The AUC in Figure 6b shows the relationship between wheat occurrence probability and mean temperature during the coldest quarter. Optimal wheat cultivation occurs at lower temperatures, particularly within 5.5°C–10°C, where the probability approaches 100%. As temperatures rise above 10°C, the probability gradually decreases, with a sharper decline beyond 16°C, indicating that higher temperatures during the coldest quarter reduce wheat suitability. This trend is reflected in the AUC, which shows a steady decline in probability as temperatures increase, particularly above 16°C. Cooler temperatures are more favourable for wheat cultivation, while higher temperatures significantly reduce its feasibility [53].

3.5.3 Wheat probability versus precipitation of wettest month

The AUC illustrating the relationship between wheat occurrence probability and the precipitation of the wettest month peaks at approximately 101.70 mm (Figure 6c). This AUC provides insights into the overall likelihood of wheat presence across various rainfall levels, highlighting the importance of precipitation in determining suitability. An elevated AUC typically suggests a strong correlation between the predictor variable (precipitation) and the outcome (wheat probability). In this case, the AUC demonstrates that increased rainfall during the wettest month significantly influences wheat occurrence probability, with a steady decline in likelihood as precipitation levels rise. This pattern suggests that moderate rainfall is more conducive to wheat occurrence, while extreme values—either too little or too much—reduce suitability (Figure 6c).

3.5.4. Wheat probability versus precipitation seasonality (coefficient of variation)

The correlation between wheat probability and precipitation seasonality, quantified by the coefficient of variation, results in an AUC of approximately 0.73%, indicating a moderate level of predictive accuracy. As shown in Figure 4e, the influence of seasonal rainfall fluctuations on the occurrence probability is evident across different levels of seasonality. The positive gradient of the curve suggests that increased precipitation seasonality is associated with a higher likelihood of wheat occurrence, implying that environments with more pronounced seasonal rainfall patterns offer more suitable conditions for wheat growth. The AUC metric further suggests that greater variability in rainfall, indicated by a higher coefficient of variation, is linked with an increased probability of wheat occurrence. This finding implies that environments with marked seasonal changes in precipitation may provide favourable conditions, potentially ensuring water availability throughout the year. The upward trend of the probability curve supports the notion that increased rainfall variability could enhance the chances of wheat survival, possibly by maintaining consistent moisture levels during critical growth phases.

3.5.5. Wheat probability versus precipitation in the coldest quarter

The investigation into the relationship between wheat occurrence probability and precipitation during the coldest quarter (Figure 6e) revealed a strong positive correlation. At low rainfall levels, the probability of wheat occurrence was less than 0.20%, increasing to 0.40% at 39.25 mm and 0.61% at 55.4 mm. The probability continued to rise, exceeding 0.90% when precipitation surpassed 80.51 mm, indicating highly favourable conditions. Beyond 100 mm, the probability approached 1.0, suggesting near-certain wheat occurrence. The AUC of 126.10 reinforces this strong correlation, highlighting that increased precipitation during this period significantly enhances the likelihood of wheat presence.

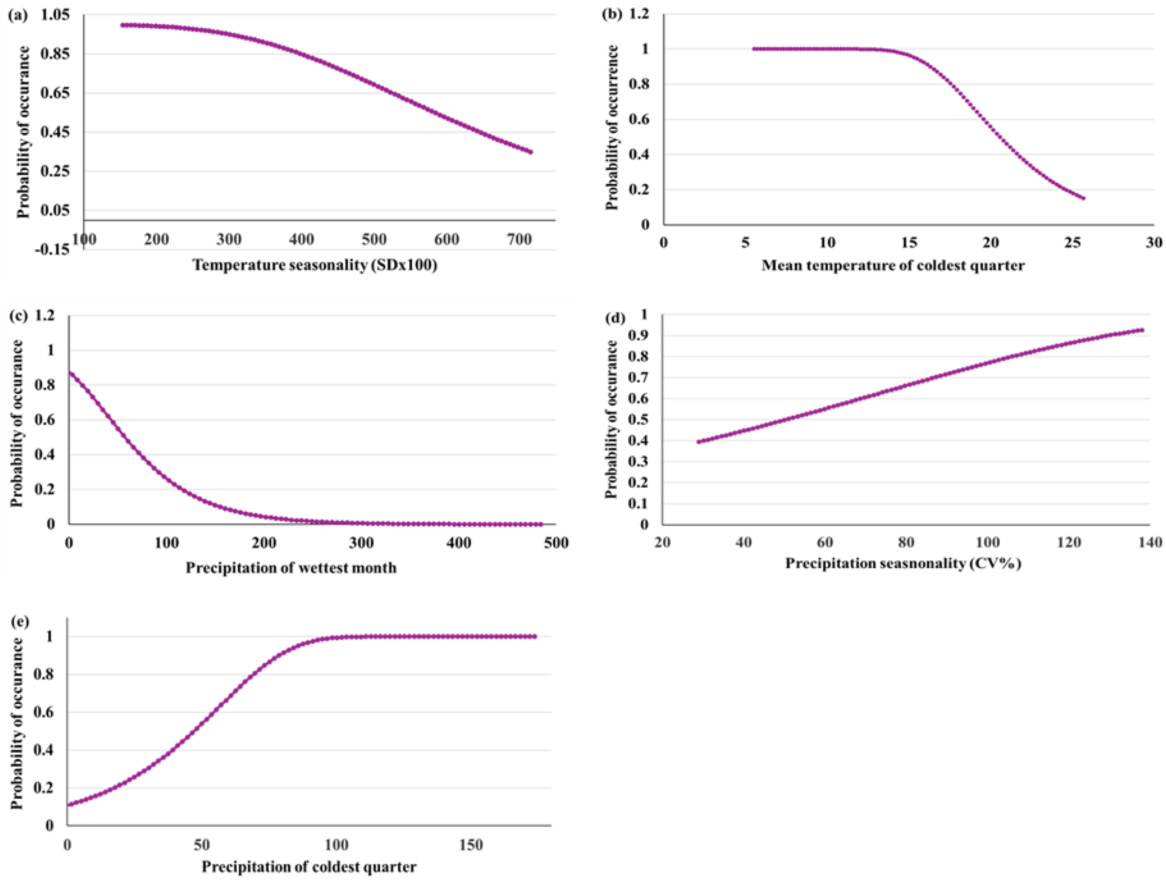

Figure 6. Wheat distribution and bioclimatic factors (1970–2020): Panels a–e show the correlations between wheat distribution and various climatic variables: temperature seasonality, mean temperature of the coldest quarter, precipitation of the wettest month, precipitation seasonality and precipitation of the coldest quarter, respectively.

3.6. Wheat versus Bioclimate Factors (2021–2040)

3.6.1. Wheat probability versus temperature seasonality (SD ×100)

The relationship between temperature seasonality and wheat suitability for 2021–2040 is illustrated by the AUC analysis (Figure 7a). During this timeframe, significant seasonal temperature variations, as indicated by higher temperature seasonality, negatively affect wheat growth [54]. Areas with minimal seasonal temperature fluctuations exhibited high wheat suitability, reaching 0.95%, suggesting that wheat thrives in climates with stable temperatures throughout the year. However, as seasonality values exceeded 300, wheat suitability declined sharply, dropping to approximately 0.44% at a seasonality of 700 mm. A critical threshold emerges

between 300 and 350 mm, where suitability remains above 0.7%. Beyond this point, suitability decreases rapidly, highlighting that greater temperature fluctuations substantially limit wheat's adaptability (Figure 7a). This identified threshold for 2021–2040 offers valuable insights for agricultural planning, underscoring regions with lower seasonality as more favourable for sustainable wheat cultivation.

3.6.2. Wheat probability versus mean temperature of the coldest quarter

As shown in Figure 7b, wheat suitability maintained its maximum probability of 1.0 until the mean temperature of the coldest quarter reached 12°C. Beyond this threshold, suitability began to decline, with a significant decrease after 17°C and reaching its lowest point above 25°C. This pattern suggests that wheat cultivation is best suited to regions where the coldest quarter temperatures remain below 12°C. Once temperatures exceed this range, especially above 17°C–18°C, the suitability for wheat cultivation decreases markedly, indicating that these warmer regions are less conducive to wheat production (Figure 7b).

3.6.3. Wheat probability versus precipitation in the wettest month

Figure 7c illustrates a clear inverse relationship between wheat suitability and rainfall during the wettest month. The probability of wheat suitability reaches its peak of 0.85% at around 3.6 mm of rainfall. However, as precipitation increases, the likelihood of suitability steadily diminishes. At 24 mm, the probability decreases to 0.73%, and at 39 mm, it further drops to 0.62%. This negative trend continues with more substantial rainfall, with the probability falling to 0.39% at 75 mm and to 0.26% at 100 mm. Beyond 150 mm of precipitation, wheat suitability drops significantly, with probabilities approaching zero once rainfall surpasses 250 mm. These findings underscore that wheat cultivation is most viable in areas with moderate rainfall during the wettest month, while higher precipitation levels reduce the likelihood of successful wheat farming [55].

3.6.4. Wheat probability versus precipitation seasonality

The relationship between wheat suitability and precipitation seasonality, as indicated by the coefficient of variation, reveals that wheat cultivation is more favourable in regions with greater rainfall variability. In areas with lower precipitation seasonality, where the coefficient of variation ranged from 28 to 40, the probability of wheat suitability remained relatively modest, at approximately 0.44% (Figure 7d). However, as the variability in precipitation increased, so did the

likelihood of wheat suitability, reaching approximately 0.87% when the coefficient of variation rose to 136. This trend suggests that wheat may perform better in regions with more pronounced seasonal fluctuations in rainfall, potentially because such conditions align water availability with critical growth stages, enhancing overall suitability for cultivation.

3.6.5. Wheat probability versus precipitation in the coldest quarter

An analysis of the correlation between wheat suitability and precipitation during the coldest quarter revealed that wheat thrives in areas with minimal rainfall during this period (Figure 7e). The likelihood of wheat suitability remained high (exceeding 0.8%) when precipitation was low (0–50 mm), suggesting that wheat flourishes in drier conditions during the coldest quarter. However, as rainfall exceeded 50 mm, the probability gradually decreased. At 100 mm, it dropped to approximately 0.5%, indicating that increased moisture in the coldest quarter is less conducive to wheat growth. Excessive moisture can lead to waterlogging, soil saturation and root diseases, all of which hinder wheat development. When precipitation exceeded 200 mm, the probability approached zero, suggesting that areas with substantial rainfall during the coldest quarter are unsuitable for wheat cultivation (Figure 7e).

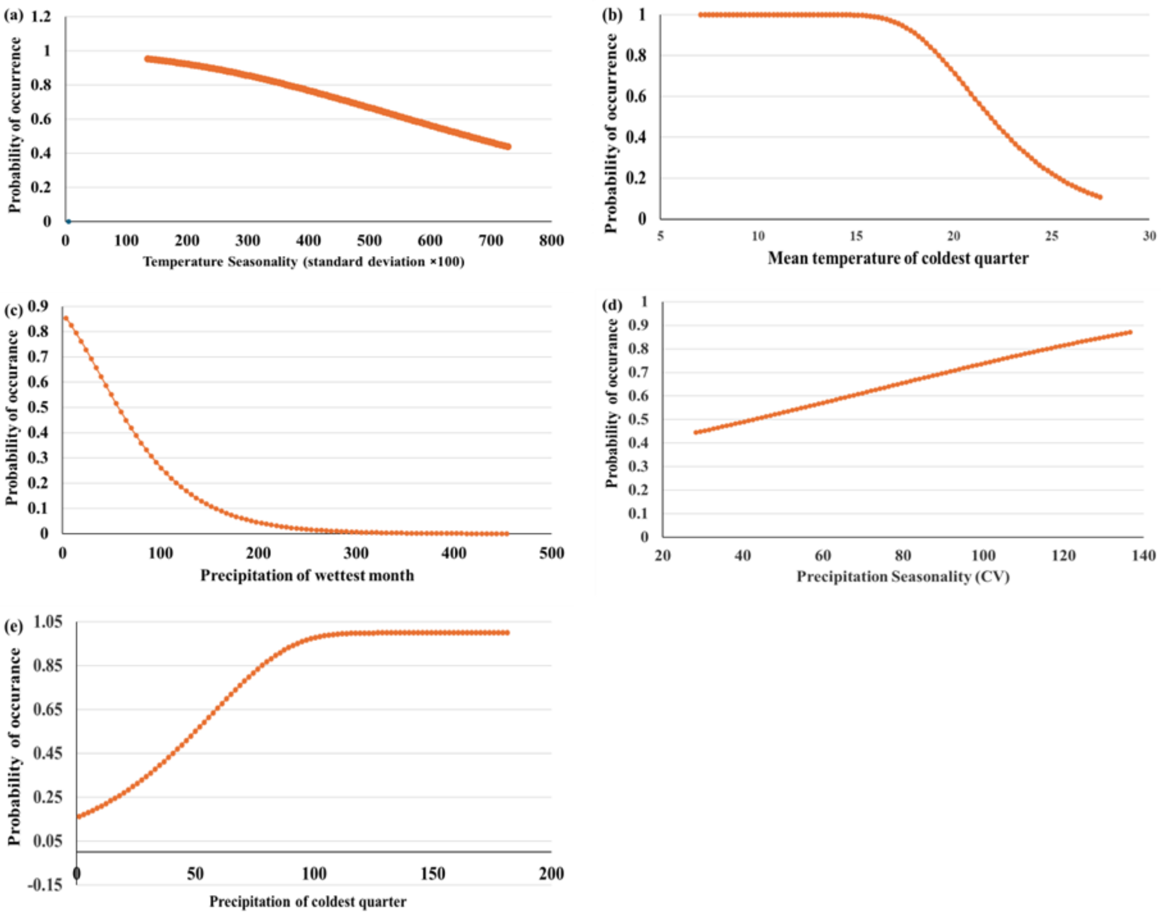

Figure 7. Wheat distribution and bioclimatic factors (2021–2040): Panels a–e show the associations between wheat distribution and key climatic parameters: temperature seasonality, average temperature of the coldest quarter, precipitation during the wettest month, precipitation seasonality and precipitation in the coldest quarter.

3.7. Wheat versus Bioclimate Factors (2041–2060)

3.7.1. Wheat probability versus temperature seasonality (SD ×100)

The projection for wheat suitability during 2041–2060, as shown in Figure 8a, indicates a decline in suitability as temperatures rise [56]. Wheat is particularly sensitive to temperature consistency, with optimal suitability occurring when seasonality ranges between 116 and 400. Suitability falls below 0.7% outside this range, marking a threshold where significant temperature fluctuations begin to hinder wheat growth. In regions where seasonality exceeds 600, suitability drops below 0.6%, suggesting that extreme temperature variations may render some areas unsuitable for wheat cultivation by 2041–2060. The MaxEnt model, through its AUC analysis, highlights the

importance of temperature stability in future wheat production and underscores the potential need for adaptive strategies in areas vulnerable to high variability.

3.7.2. Wheat probability versus mean temperature of the coldest quarter

The relationship between wheat probability and the average temperature of the coldest quarter shows optimal conditions between 9°C and 13.75°C, with 100% probability (Figure 8b). As temperatures rise above 13.75°C, suitability declines. Wheat can still grow at 14°C–15°C, but with reduced suitability. When temperatures exceed 19°C, the probability falls below 0.95%, dropping further to 0.60% at 22.4°C and under 0.30% above 26°C. Areas with cold-quarter temperatures exceeding 28°C are highly unsuitable for wheat cultivation [57].

3.7.3. Wheat probability versus precipitation in the wettest month

A clear trend emerges between precipitation in the wettest month and wheat suitability, with increasing rainfall steadily reducing favourable conditions (Figure 8c). Optimal suitability occurs with minimal or no precipitation, where levels between 0 and 8 mm correspond to a probability exceeding 0.80%. As precipitation increases, suitability declines, dropping from 0.77% at 18 mm to 0.57% at 49 mm. Beyond 50 mm, the decline becomes more pronounced, reaching 0.27% at 100 mm and further decreasing at higher levels. Rainfall above 200 mm results in extremely low probabilities, indicating unsuitable conditions for wheat cultivation. This suggests that areas with lower rainfall in the wettest month are more conducive to wheat growth.

3.7.3. Wheat probability versus precipitation seasonality

The data revealed a clear positive correlation between precipitation seasonality and wheat suitability (Figure 8d). The likelihood of wheat suitability increased with greater seasonal rainfall variability, suggesting that areas with more pronounced precipitation fluctuations are more conducive to wheat growth. In regions with moderate precipitation seasonality (30–60 mm), the probability gradually rose from 0.41% to 0.55%. When seasonality exceeded 60 mm, the probability increased more sharply, reaching 0.65% at 80 mm and 0.70% at 90 mm. These findings indicate that areas with higher seasonal precipitation fluctuations are more suitable for wheat cultivation.

The probability continued to rise with higher levels of precipitation seasonality. For instance, when seasonality reached 100 mm, the probability was roughly 0.79%, and it kept increasing as seasonality rose, reaching 0.85% beyond 120 mm and peaking at 0.89% when seasonality values were around 134 mm (Figure 8d), indicating that areas with the most pronounced precipitation seasonality are expected to be the most conducive to the modelled scenario. Overall, the findings indicated that increased precipitation seasonality is associated with a higher probability of suitability. Areas with more significant seasonal rainfall fluctuations became increasingly favourable, with the highest probabilities observed at the upper extremes of the seasonality range. This implies that the modelled conditions are most suited to environments where rainfall is distributed unevenly throughout the year.

3.7.5. Wheat probability versus precipitation in the coldest quarter

A distinct positive association between precipitation during the coldest quarter and wheat probability is evident (Figure 8e). In areas with very low rainfall, such as 6 mm, the probability began at 0.20%, indicating that locations with minimal precipitation during the coldest quarter were less conducive to the modelled conditions. As rainfall increased slightly, the probability rose gradually but remained under 0.3% until precipitation reached approximately 15 mm, suggesting that areas with low rainfall remained relatively unfavourable.

As precipitation levels increased from 15 mm to 50 mm, a significant improvement in probability was observed, with values rising from 0.32% to 0.55%. This indicates that moderate rainfall amounts correspond to a higher likelihood of favourable conditions, signalling a trend towards greater suitability. This range of precipitation appears more advantageous for the modelled scenario, as the probability surpassed the 0.5% mark (Figure 8e). The probability steadily increased as rainfall exceeded 50 mm. For instance, it reached 0.62% at 60 mm and 0.80% at 80 mm, suggesting that higher precipitation during the coldest quarter enhances suitability, indicating a strong positive correlation between rainfall and habitability within this range. The probability consistently exceeded 0.90% when precipitation exceeded 100 mm. For instance, at 99 mm, the probability was 0.92%, and it continued to rise rapidly thereafter. As precipitation reached and surpassed 150 mm, the probability approached 100%, signalling near-ideal suitability.

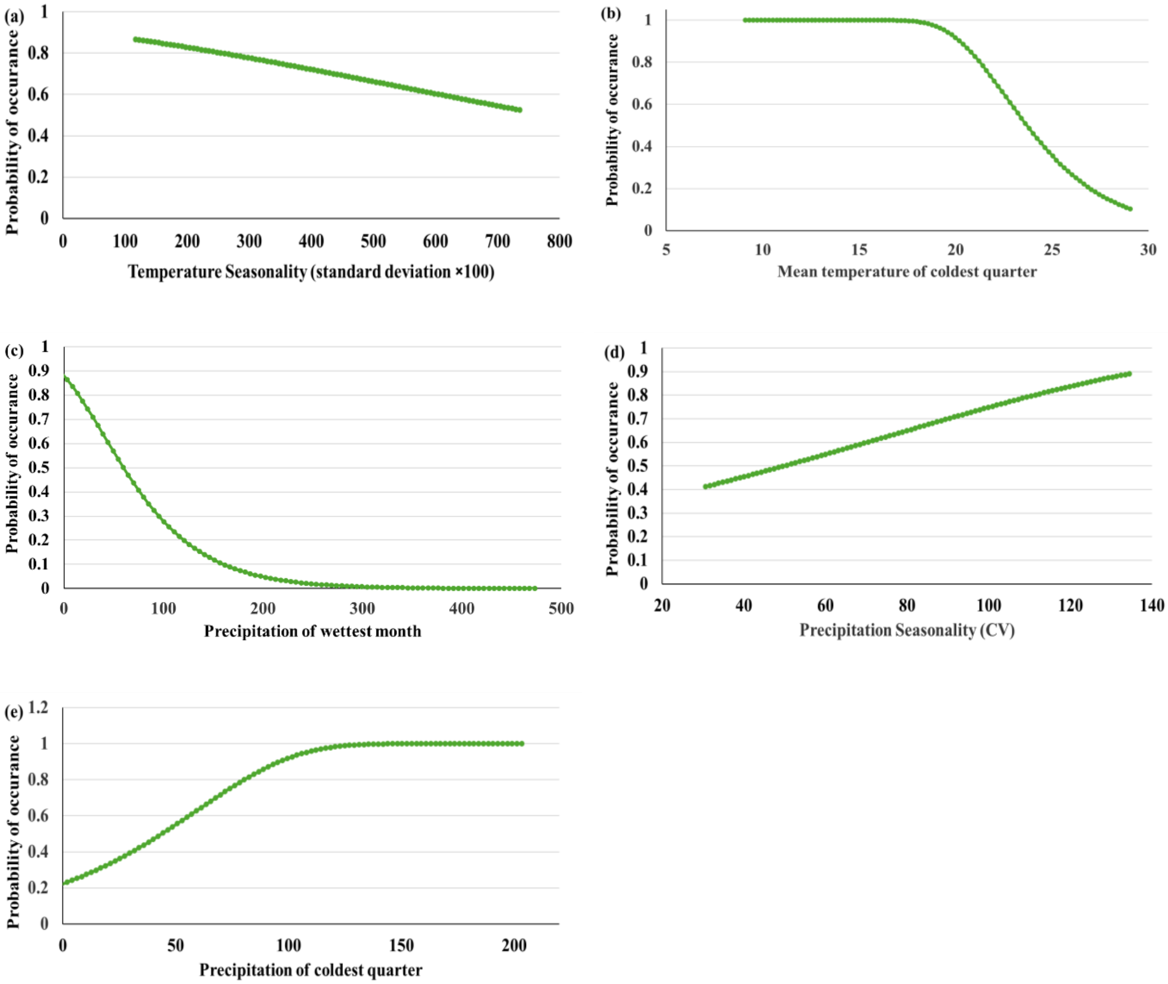

Figure 8. Wheat suitability projection during 2041–2060.

3.8. Wheat versus Bioclimate Factors (2061–2080)

3.8.1. Wheat probability versus temperature seasonality (SD ×100)

The analysis of temperature seasonality and wheat suitability for 2061–2080 reveals a clear inverse relationship (Figure 9a). As temperatures rise, the likelihood of suitable conditions for wheat cultivation steadily diminishes. In areas with lower temperature variability (approximately 108), the probability of wheat suitability peaks at 0.96%, suggesting optimal growing conditions. This elevated probability remains relatively stable for temperature seasonality values up to approximately 250, with the likelihood generally exceeding 0.88%.

When temperature seasonality exceeds 250 (SD ×100), the likelihood of wheat suitability begins to decrease more noticeably. At 300, the probability remains relatively high at 0.86%, but it

continues to decline thereafter. Once temperature seasonality reaches 400, the probability drops to 0.75%, indicating that more extreme temperature fluctuations start to negatively affect wheat cultivation.

As temperature seasonality surpasses 450, a more pronounced reduction is observed. At this point, the probability decreases to 0.72%, further diminishing to around 0.66% at 500. This suggests that areas experiencing greater temperature fluctuations are progressively less suitable for wheat production. The most significant decline occurs when seasonality exceeds 600, with the probability plummeting to 0.56% and continuing to decrease as seasonality intensifies. When seasonality reaches 750°C, the probability decreases to 0.45%, indicating that regions with extreme temperature variations will face considerable challenges in sustaining wheat crops.

Our research suggests that wheat grows best in areas with stable temperatures where successful cultivation is highly probable. As temperature fluctuations become more pronounced, particularly when seasonality surpasses 500 (SD ×100), the suitability for wheat farming decreases significantly. This trend implies that wheat production will remain more favourable in regions with consistent thermal conditions, while areas with greater temperature variability will struggle to sustain wheat crops during 2061–2080 (Figure 9a).

3.8.2. Wheat probability versus mean temperature of the coldest quarter

The analysis reveals a strong correlation between the mean temperature of the coldest quarter and the likelihood of wheat suitability. Optimal conditions for wheat growth were observed when the average temperature was 10.6°C–15.8°C, with a 100% probability of suitability (Figure 9b). These cooler temperatures during the coldest quarter provide ideal conditions for wheat cultivation, making these areas highly appropriate for growth.

As the average temperature exceeds 16°C, the likelihood of wheat suitability begins to decrease slightly. For instance, at 17°C, the probability remains high but drops slightly to 0.99. This gradual decline, although initially modest, signals a steady reduction in wheat suitability as temperatures rise, even though conditions still favour wheat growth (Figure 9b). The decline becomes more pronounced as the average temperature approaches 20°C. At 20.72°C, the probability decreases to 0.97% and continues to decline as temperatures rise further. When the temperature reaches 21.33°C, the probability drops to 0.94%, demonstrating a consistent reduction in wheat suitability

as temperatures during the coldest quarter exceed 20°C. Despite the suitability remaining relatively high, the observed pattern indicates that elevated temperatures in the cold quarter begin to negatively affect wheat growth (Figure 9b).

The suitability of wheat cultivation sharply declined as temperatures exceeded 22°C. The probability decreased to 0.84% at 22.54°C and to 0.72% at 23.56°C, reflecting a significant reduction in wheat's growth potential. This suggests that areas with cold-quarter temperatures above 22°C are increasingly unsuitable for wheat cultivation. The most notable decline occurred at temperatures above 25°C, with the probability dropping to 0.43% at 25.78°C and 0.32% at 26.8°C. Areas with cold-quarter mean temperatures exceeding 28°C showed extremely low probabilities, falling below 0.2%, making these regions highly unfavourable for wheat production. Thus, during 2061–2080, wheat cultivation will be most viable in cooler regions where mean cold-quarter temperatures fall between 10.6°C and 15.8°C. Wheat suitability declined gradually as temperatures rose above 16°C, with more pronounced reductions after 20°C. Regions with cold-quarter temperatures above 25°C became markedly less suitable, with probabilities near zero for supporting wheat growth (Figure 9b). This analysis highlights the essential role of cold-quarter temperatures in determining wheat cultivation potential in the future.

3.8.3. Wheat probability versus precipitation during the wettest month

The relationship between precipitation during the wettest month and wheat suitability showed a clear pattern. As precipitation increased, the probability of suitable conditions for wheat cultivation declined gradually. For example, when precipitation reached 20 mm, the probability dropped to 0.83%, and by 35 mm, it decreased further to 0.70%. This suggests that although areas with moderate rainfall still retain some suitability for wheat, the likelihood of suitability steadily diminishes as precipitation exceeds 20 mm (Figure 9c).

A more noticeable decline was observed when precipitation exceeded 50 mm. At 50.7 mm, the probability decreased to 0.56%, and by 66 mm, it dropped to 0.43%. This indicates that areas with higher precipitation during the wettest month become increasingly less suitable for wheat cultivation, with conditions turning less favourable as rainfall increases.

Once precipitation surpassed 80 mm, the decline in suitability became more pronounced. At 81 mm, the probability dropped to 0.32%, and by 96.7 mm, it further decreased to 0.23%. This sharp

reduction suggests that regions with heavy rainfall, particularly above 80 mm, are much less suitable for wheat cultivation. In areas with extreme precipitation, particularly above 150 mm, the probability approaches zero. For instance, at 150 mm, the probability is just 0.07%, and as rainfall continues to rise, the probability falls even further. At 250 mm, the probability is almost zero, indicating that regions with excessive rainfall during the wettest month are unsuitable for wheat production.

3.8.4. Wheat probability versus precipitation in the coldest quarter

The correlation between rainfall during the coldest quarter and the likelihood of wheat suitability showed a clear positive trend as precipitation levels increased. In regions with lower rainfall (approximately 31–40 mm), the probability of wheat suitability remained relatively modest, fluctuating between 0.32% and 0.37%. This suggests that areas experiencing minimal precipitation during the coldest quarter possess moderate potential for wheat cultivation, but suitability improves as rainfall increases.

The probability of wheat suitability demonstrated a steady increase as precipitation rose from 40 mm to 70 mm. For example, the likelihood increased from 0.40% at 45 mm to 0.57% at 70 mm. This pattern indicates that regions with higher precipitation during the coldest quarter become progressively more suitable for wheat farming, surpassing the key 0.50% threshold and signalling a marked improvement in conditions for wheat growth.

When precipitation reached between 70 mm and 100 mm, the likelihood of wheat suitability rose significantly. At 80 mm, the probability was 0.65%, and at 100 mm, it increased to 0.79%. This indicates that areas with higher rainfall during the coldest quarter are substantially more favourable for wheat cultivation, with probabilities approaching 0.80%, reflecting a strong potential for successful wheat farming.

Beyond 100 mm of precipitation, the probability continued to rise, peaking at 120 mm. For instance, at 110 mm, the probability was 0.85%, and at 120 mm, it reached 0.90%. This suggests that regions with moderate to high rainfall during the coldest quarter provide optimal conditions for wheat cultivation, with probabilities nearing their maximum.

For precipitation levels exceeding 120 mm, the probability remained high, exceeding 0.90%. At 130 mm, the probability increased to 0.94%, and at 132 mm, it rose further to 0.95%. This plateau

indicates that areas receiving abundant rainfall during the coldest quarter offer near-ideal conditions for wheat cultivation, with minimal variation in suitability once precipitation surpasses 120 mm [58].

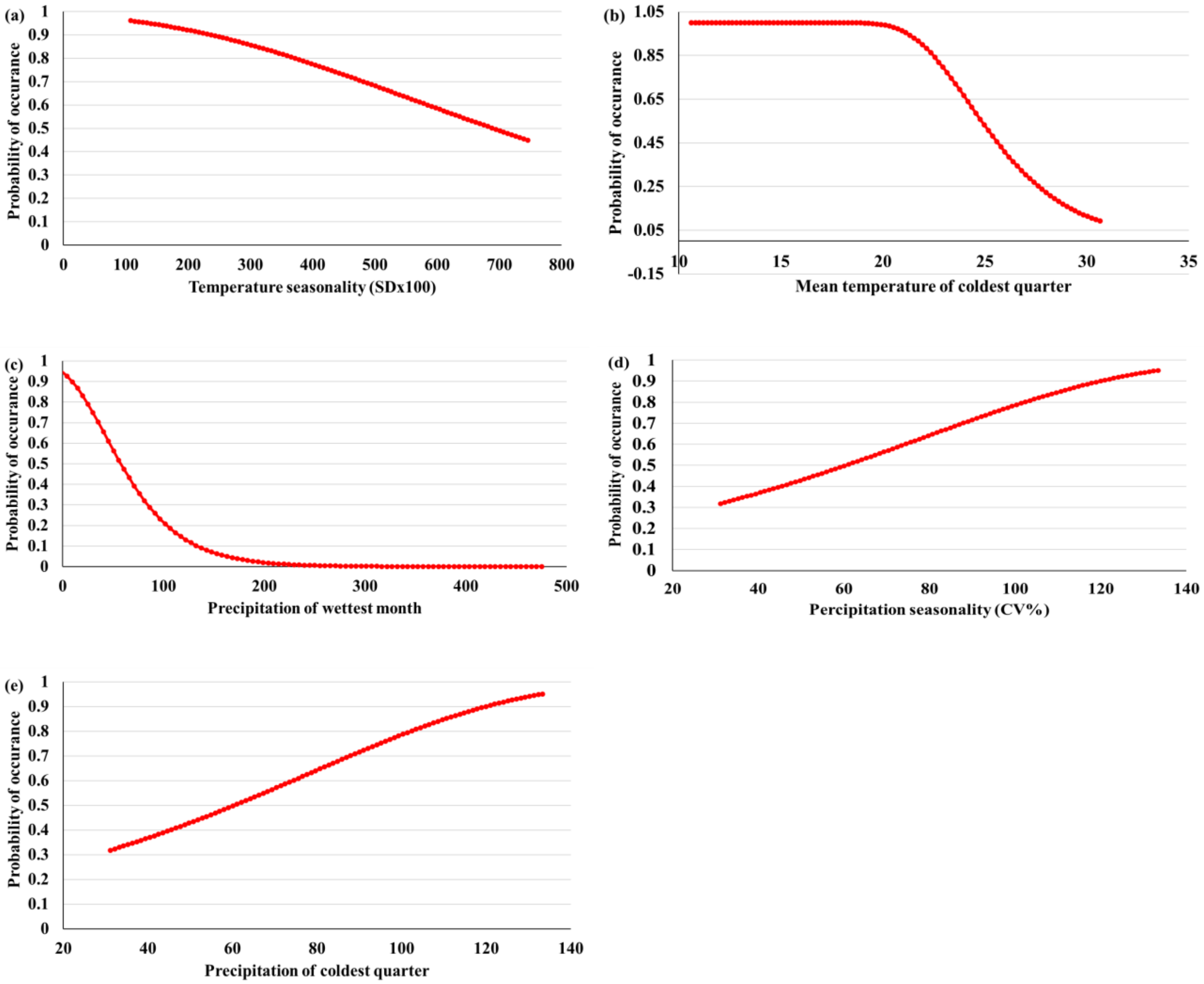

Figure 9. Temperature seasonality and wheat suitability analysis for 2061–2080.

3.9 Further Research

Future research could leverage advanced suitability, simulation and predictive models, incorporating factors such as cultural practices, socioeconomic influences, ecological elements and meteorological characteristics. These models would help identify the best combinations of features for determining optimal wheat cultivation sites and forecast wheat distribution under current conditions, guiding management and resource allocation.

4. Conclusion

This study highlights the complex relationships between environmental factors and wheat cultivation, offering insights into the impacts of climate and precipitation variability. Using MaxEnt modelling, we found that wheat thrives in regions with stable temperature seasonality, moderate cold-season temperatures (below 12°C) and low-to-moderate rainfall. Wheat suitability decreases when temperature seasonality exceeds 300, cold-season temperatures exceed 17°C–18°C, or precipitation during the coldest quarter exceeds 100 mm. Similarly, high rainfall during the wettest month (above 75 mm) reduces wheat viability. Wheat is more adaptable to areas with higher precipitation seasonality, likely due to better water availability during key growth periods. These findings have significant implications for agricultural planning, climate resilience, and the development of climate-resilient wheat varieties. Future research should integrate soil properties, land use, and long-term climate projections to refine models and build a comprehensive framework for climate-resilient agriculture.

Acknowledgements:

The authors gratefully thank The Ministry of Agriculture Fisheries Wealth & Water Resources for providing the facilities for supporting the related research.

Author contributions:

KA and AL contributed to study conception and design. Material preparation was performed by KA and Al. Data collection, analysis and evaluation of climate change and its impact on wheat distribution in semi-arid countries were performed by: KA and AL. Critical data review was performed by KA and Al . The first draft of the manuscript was written by KA and AL, and all authors commented on previous versions of the manuscript. All authors read and approved of the final manuscript.

Funding: Not applicable.

Data availability: Not applicable.

Declarations

Competing interests: The authors declare no competing interests.

References:

1. Hayat A, Tahir M. Natural resources volatility and economic growth: evidence from the resource-rich region. J Risk Financ Manag. 2021;14: 84.

2. Victor R. Biodiversity conservation and sustainable development: A case study of Oman's National Biodiversity Strategy and Action Plan. Conserving Biodiversity in Arid Regions: Best Practices in Developing Nations. Springer; 2003. pp. 461–480.

3. Paymard P, Bannayan M, Haghighi RS. Analysis of the climate change effect on wheat production systems and investigate the potential of management strategies. Nat Hazards. 2018;91: 1237–1255.

4. Ahmed M, Choudri BS. Climate change in Oman: Current knowledge and way forward. Educ Bus Soc Contemp Middle East Issues. 2012;5: 228–236. doi:10.1108/17537981211284416

5. Fawzy S, Osman AI, Doran J, Rooney DW. Strategies for mitigation of climate change: a review. Environ Chem Lett. 2020;18: 2069–2094.

6. Malhi GS, Kaur M, Kaushik P. Impact of climate change on agriculture and its mitigation strategies: A review. Sustainability. 2021;13: 1318.

7. Tol RSJ. The economic impacts of climate change. Rev Environ Econ policy. 2018.

8. Hatfield JL, Antle J, Garrett KA, Izaurralde RC, Mader T, Marshall E, et al. Indicators of climate change in agricultural systems. Clim Change. 2020;163: 1719–1732.

9. Bajwa AA, Farooq M, Al-Sadi AM, Nawaz A, Jabran K, Siddique KHM. Impact of climate change on biology and management of wheat pests. Crop Prot. 2020;137: 105304.

10. Farooq M. Wheat production in Oman: experiences, challenges and opportunities. J Agric Mar Sci [JAMS]. 2022;27: 1–15.

11. Zahra N, Hafeez MB, Wahid A, Al Masruri MH, Ullah A, Siddique KHM, et al. Impact of climate change on wheat grain composition and quality. J Sci Food Agric. 2023;103: 2745–2751.

12. Filatenko AA, Hammer K. Wheat landraces from Oman: A botanical analysis. Emirates J

Food Agric. 2014;26: 119–137.

13. Al Lawati AH, Nadaf SK, AlSaady NA, Al Hinai SA, Almamari AR, Al Maawali AA. Genetic diversity of Omani barley (Hordeum vulgare L.) germplasm. Open Agric. 2021;6: 628–639.

14. Alsafadi K, Bi S, Bashir B, Alsalman A, Srivastava AK. Future scenarios of bioclimatic viticulture indices in the eastern Mediterranean: Insights into sustainable vineyard management in a changing climate. Sustainability. 2023;15: 11740.

15. Burri NM, Weatherl R, Moeck C, Schirmer M. A review of threats to groundwater quality in the anthropocene. Sci Total Environ. 2019;684: 136–154. doi:https://doi.org/10.1016/j.scitotenv.2019.05.236

16. Eliades M, Michaelides S, Evagorou E, Fotiou K, Fragkos K, Leventis G, et al. Earth Observation in the EMMENA Region: Scoping Review of Current Applications and Knowledge Gaps. Remote Sens. 2023;15: 4202.

17. Tang X, Yuan Y, Li X, Zhang J. Maximum entropy modeling to predict the impact of climate change on pine wilt disease in China. Front Plant Sci. 2021;12: 652500.

18. Ali S, Makanda TA, Umair M, Ni J. MaxEnt model strategies to studying current and future potential land suitability dynamics of wheat, soybean and rice cultivation under climatic change scenarios in East Asia. PLoS One. 2023;18: e0296182.

19. Yue Y, Zhang P, Shang Y. The potential global distribution and dynamics of wheat under multiple climate change scenarios. Sci Total Environ. 2019;688: 1308–1318.

20. Khubaib N, Asad SA, Khalil T, Baig A, Atif S, Umar M, et al. Predicting areas suitable for wheat and maize cultivation under future climate change scenarios in Pakistan. Clim Res. 2021;83: 15–25.

21. Zhao J, Yang J, Huang R, Xie H, Qin X, Hu Y. Estimating evapotranspiration and drought dynamics of winter wheat under climate change: A case study in Huang-Huai-Hai region, China. Sci Total Environ. 2024;949: 175114.

22. Al-Sarmi S, Al-Yahyai S, Al-Maskari J, Charabi Y, Choudri BS. Recent Observed

Climate Change Over Oman. Springer Water. Springer Nature; 2017. pp. 89–100. doi:10.1007/978-3-319-51856-5_6

23. Coll J. Recent trends in temperature and precipitation in Al Jabal Al Akhdar, Sultanate of Oman, and the implications for future climate change. J Earth Sci Clim Chang. 2015;6.

24. Farooq MS, Uzair M, Raza A, Habib M, Xu Y, Yousuf M, et al. Uncovering the research gaps to alleviate the negative impacts of climate change on food security: a review. Front Plant Sci. 2022;13: 927535.

25. Oke J, Akinkunmi WB, Etebefia SO. Use of correlation, tolerance and variance inflation factor for multicollinearity test. GSJ. 2019;7: 652–659.

26. Tay R. Correlation, variance inflation and multicollinearity in regression model. J East Asia Soc Transp Stud. 2017;12: 2006–2015.

27. Shrestha N. Detecting multicollinearity in regression analysis. Am J Appl Math Stat. 2020;8: 39–42.

28. Duan R-Y, Kong X-Q, Huang M-Y, Fan W-Y, Wang Z-G. The predictive performance and stability of six species distribution models. PLoS One. 2014;9: e112764.

29. Merow C, Smith MJ, Silander Jr JA. A practical guide to MaxEnt for modeling species' distributions: what it does, and why inputs and settings matter. Ecography (Cop). 2013;36: 1058–1069.

30. Li X, Wang Y. Applying various algorithms for species distribution modelling. Integr Zool. 2013;8: 124–135.

31. Elith J, Phillips SJ, Hastie T, Dudík M, Chee YE, Yates CJ. A statistical explanation of MaxEnt for ecologists. Divers Distrib. 2011;17: 43–57.

32. Yackulic CB, Chandler R, Zipkin EF, Royle JA, Nichols JD, Campbell Grant EH, et al. Presence-only modelling using MAXENT: when can we trust the inferences? Methods Ecol Evol. 2013;4: 236–243.

33. Halvorsen R, Mazzoni S, Dirksen JW, Næsset E, Gobakken T, Ohlson M. How important are choice of model selection method and spatial autocorrelation of presence data for

distribution modelling by MaxEnt? Ecol Modell. 2016;328: 108–118.

34. Zeng Y, Low BW, Yeo DCJ. Novel methods to select environmental variables in MaxEnt: A case study using invasive crayfish. Ecol Modell. 2016;341: 5–13.

35. Faraggi D, Reiser B. Estimation of the area under the ROC curve. Stat Med. 2002;21: 3093–3106.

36. Chen P, Zhu Y. A new method for winter wheat critical nitrogen curve determination. Agron J. 2013;105: 1839–1846.

37. Justes E, Mary B, Meynard J-M, Machet J-M, Thelier-Huché L. Determination of a critical nitrogen dilution curve for winter wheat crops. Ann Bot. 1994;74: 397–407.

38. Yang G, Li X, Liu P, Yao X, Zhu Y, Cao W, et al. Automated in-season mapping of winter wheat in China with training data generation and model transfer. ISPRS J Photogramm Remote Sens. 2023;202: 422–438.

39. Talluri R, Shete S. Using the weighted area under the net benefit curve for decision curve analysis. BMC Med Inform Decis Mak. 2016;16: 1–9.

40. Kyriazos T, Poga M. Dealing with multicollinearity in factor analysis: the problem, detections, and solutions. Open J Stat. 2023;13: 404–424.

41. Al Kindi KM, Kwan P, Andrew NR, Welch M. Modelling the potential effects of climate factors on Dubas bug (Ommatissus lybicus) presence/absence and its infestation rate: A case study from Oman. Pest Manag Sci. 2019;75: 3039–3049. doi:https://doi.org/10.1002/ps.5420

42. Guo X, Zhang P, Yue Y. Prediction of global wheat cultivation distribution under climate change and socioeconomic development. Sci Total Environ. 2024;919: 170481. doi:https://doi.org/10.1016/j.scitotenv.2024.170481

43. Jiang T, Wang B, Xu X, Cao Y, Li Liu D, He L, et al. Identifying sources of uncertainty in wheat production projections with consideration of crop climatic suitability under future climate. Agric For Meteorol. 2022;319: 108933.

44. Al-Kindi KM, Alqurashi AF, Al-Ghafri A, Power D. Assessing the Impact of Land Use

and Land Cover Changes on Aflaj Systems over a 36-Year Period. Remote Sensing. 2023. doi:10.3390/rs15071787

45. Serrano J, Shahidian S, Marques da Silva J, Paixão L, Carreira E, Pereira A, et al. Climate changes challenges to the management of Mediterranean Montado ecosystem: Perspectives for use of precision agriculture technologies. Agronomy. 2020;10: 218.

46. Anwar MR, O'Leary GJ, Rab MA, Fisher PD, Armstrong RD. Advances in precision agriculture in south-eastern Australia. V. Effect of seasonal conditions on wheat and barley yield response to applied nitrogen across management zones. Crop Pasture Sci. 2009;60: 901–911.

47. Fang Q, Zhang X, Shao L, Chen S, Sun H. Assessing the performance of different irrigation systems on winter wheat under limited water supply. Agric water Manag. 2018;196: 133–143.

48. Heng LK, Asseng S, Mejahed K, Rusan M. Optimizing wheat productivity in two rain-fed environments of the West Asia–North Africa region using a simulation model. Eur J Agron. 2007;26: 121–129.

49. Nguyen TT, Grote U, Neubacher F, Do MH, Paudel GP. Security risks from climate change and environmental degradation: Implications for sustainable land use transformation in the Global South. Curr Opin Environ Sustain. 2023;63: 101322.

50. Sundström JF, Albihn A, Boqvist S, Ljungvall K, Marstorp H, Martiin C, et al. Future threats to agricultural food production posed by environmental degradation, climate change, and animal and plant diseases–a risk analysis in three economic and climate settings. Food Secur. 2014;6: 201–215.

51. Al-Kindi KM. Assessing the environmental factors affecting the sustainability of Aini Falaj system. PLoS One. 2024;19: e0301832.

52. Huang J, Wang S, Guo Y, Chen J, Yao Y, Chen D, et al. Hysteresis between winter wheat canopy temperature and atmospheric temperature and its driving factors. Plant Soil. 2024;499: 55–71.

53. Kawasaki K. Two harvests are better than one: double cropping as a strategy for climate

change adaptation. Am J Agric Econ. 2019;101: 172–192.

54. You L, Rosegrant MW, Wood S, Sun D. Impact of growing season temperature on wheat productivity in China. Agric For Meteorol. 2009;149: 1009–1014.

55. Sassenrath GF, Farney J, Lollato R. These results suggest that wheat cultivation flourishes in areas with moderate rainfall during the wettest month, while higher precipitation levels are less conducive to successful wheat production. Crop Forage Turfgrass Manag. 2019;5: 1–10.

56. Nehbandani A, Alizadeh-Dehkordi P, Dadrasi A, Filippi P, Hajjarpoor A. Global Suitability Analysis of Current and Future Climates for Rainfed Wheat Production. Int J Plant Prod. 2023;17: 579–592.

57. Lloret F, Escudero A, Iriondo JM, Martínez-Vilalta J, Valladares F. Extreme climatic events and vegetation: the role of stabilizing processes. Glob Chang Biol. 2012;18: 797–805.

58. Parmesan C, Root TL, Willig MR. Impacts of extreme weather and climate on terrestrial biota. Bull Am Meteorol Soc. 2000;81: 443–450.
