# Supplementary material for: Climate change and its impact on wheat distribution in semi-arid ecosystems: A case study from the Sultanate of Oman
Source: PLoS One. 2025 Jun 17;20(6):e0326198. doi: 10.1371/journal.pone.0326198 (PMC12173393; doi:10.1371/journal.pone.0326198)
Supplement: Attachment — Submitted filename: Respone to Review comments.docx [file pone.0326198.s006.docx]

Dear Editor and Reviewer’s,

We wish to convey our heartfelt gratitude for the detailed and insightful feedback on our manuscript. We are genuinely thankful for the time and effort you dedicated to offering such comprehensive insights. Below, you will find our responses to your comments, along with the revisions we have made to improve the clarity, precision, and originality of our research.

Regards,

Al-Kindi

Review Comments to the Author

Reviewer #2: After a re-review, I'm satisfied with the authors' responses. The revised manuscript and the authors' letter address my primary concerns. I have no other comments and I have enjoyed reading the revised manuscript. I found the manuscript worthy of publication. Therefore, I recommend publishing this paper in the current form. I thank the authors and congratulate them.

We sincerely appreciate the time and effort you invested in reviewing our manuscript. Your constructive feedback has been crucial in improving the quality of our work. We are delighted to learn that you found our revisions satisfactory and that our responses adequately addressed your primary concerns. Your positive comments and recommendation for publication are incredibly motivating. Thank you once again for your thoughtful review and kind words. We greatly value your support and significant contribution to this research.

Reviewer #3: "Climate Change and its Impact on Wheat Distribution in Semi-arid Ecosystems: A case study from the Sultanate of Oman" This study investigates the relationships between wheat distribution and a set of predetermined climatic factors (for both current and future scenarios). After reviewing the revised manuscript and the authors' responses to the reviewers' comments, the work appears well-designed in its current form, with results presented clearly and logically. The study is now recommended for acceptance pending the incorporation of the following changes.

Dear Reviewer,

We are truly grateful for your insightful review and the positive remarks regarding our manuscript. It is gratifying to know that you consider the study to be well-structured and that the revisions have enhanced the clarity and logical flow of the results. We recognize your suggestions for additional modifications and will thoughtfully integrate them to further improve the manuscript's quality. Your valuable insights have significantly contributed to the refinement of our work, and we appreciate the time and effort you have dedicated to reviewing our study.

Discussion:
If the journal’s guidelines permit, I suggest tidying up the Discussion section by separating it into an independent section. Within this section, you could describe the relative contributions of individual climatic variables to the target species’ distribution. Additionally, I recommend including two subsections, addressing the limitations and implications of the modeling technique. For this, the following references conducted in the Middle East may be relevant. In any case, adding few sentences on the limitations and implications of the study is required.
<https://doi.org/10.1016/j.ecoinf.2022.101930>
<https://doi.org/10.1007/s10661-024-12438-z>
<https://www.mdpi.com/2071-1050/14/21/14621>
<https://doi.org/10.1016/j.jnc.2023.126505>

Dear Reviewer,

We value your feedback. Based on your comments and suggestions, we have incorporated sentences into the revised manuscript that clarify the relative contributions of individual climate variables to the distribution of the target species. Please refer to lines 633-644. Furthermore, we have added a new section to the discussion, which explores the implications and limitations of the modeling technique. See lines 645-657 for this addition. Consequently, we have included more pertinent citations in the revised manuscript.

Reviewer #4: This study investigates the influence of temperature and precipitation on wheat distribution in the Sultanate of Oman, classifying regions into varying suitability levels. The findings offer significant insights and practical guidance for wheat cultivation in the area. However, several issues need to be addressed, as outlined below:

1. The abstract requires substantial refinement and should be rephrased to align more closely with the central theme and key findings of the study. For instance, the first sentence of the abstract is identical to the opening sentence of the introduction, which is not permissible in academic writing. The abstract should succinctly encapsulate the primary objectives, methodologies, results, and conclusions of the research.

Dear Reviewer,

Thank you very much for your feedback. We have updated the abstract in the revised version of the manuscript. Please see the abstract section in the revised version.

2. The WorldClim database comprises 19 bioclimatic variables. A clear rationale and justification for the selection of the 9 variables used in this study, as well as the exclusion of the remaining 10, should be provided. This will enhance transparency regarding the criteria employed in the variable selection process.

Dear Reviewer,

We appreciate your feedback. We have deliberated on the choice of the WorldClim database. Please refer to lines 143-149. Additionally, new sentences have been incorporated into the updated manuscript. Please check lines 137-139.

3. Topographic characteristics, hydrological distribution, and soil properties are critical determinants of plant growth and distribution. It is essential to explain why these factors were not incorporated into the model. Their inclusion could offer a more holistic assessment of wheat suitability and improve the robustness of the findings.

Dear Reviewer,

We appreciate your comment. We acknowledge that terrain features, water resource distribution, and soil properties are key factors affecting plant growth and distribution. These aspects influence water availability, nutrient dynamics, and microclimatic conditions, all of which are essential for wheat cultivation. Elements such as elevation, slope, and aspect impact temperature variations and soil moisture retention, while hydrological factors like groundwater access and drainage patterns are crucial for sustaining crop productivity. Furthermore, soil characteristics, including texture, organic matter content, and pH, directly affect root development and nutrient uptake. Although these factors are highly relevant for evaluating wheat suitability, they were not directly included in our modeling approach, which focused primarily on climatic variables due to their dominant influence on crop suitability in future climate scenarios. Given the regional scope of our analysis and the limitations in the spatial resolution of certain soil and hydrological datasets, we prioritized climate-based predictors to ensure consistency in the model's application. Nonetheless, recognizing their significance, we recommend that future research incorporate these additional parameters to provide a more comprehensive assessment of wheat cultivation potential. This information has already been mentioned in our manuscript. Please refer to the Future Research Section (lines 620-631).

4. The classification of suitability into five categories—extremely high, high, medium, low, and extremely low—requires clarification. Please provide the specific criteria or methodological framework used to define these categories. This will facilitate a clearer understanding of how the suitability thresholds were established and interpreted within the context of the study.

Dear Reviewer,

We sincerely value your input. We have added several sentences to the revised manuscript, which can be found on lines 176-184 and lines 236-238.
